# Selective NMR observation of the SEI–metal interface by dynamic nuclear polarisation from lithium metal

**DOI:** 10.1038/s41467-020-16114-x

**Published:** 2020-05-06

**Authors:** Michael A. Hope, Bernardine L. D. Rinkel, Anna B. Gunnarsdóttir, Katharina Märker, Svetlana Menkin, Subhradip Paul, Ivan V. Sergeyev, Clare P. Grey

**Affiliations:** 1 Department of Chemistry, University of Cambridge, Lensfield Road, Cambridge, CB2 1EW UK; 2 School of Physics and Astronomy, University of Nottingham, University Park, Nottingham, NG7 2RD UK; 3 Bruker Biospin Corp., 15 Fortune Drive, Billerica, MA 01821 USA

**Keywords:** Solid-state NMR, Batteries

## Abstract

While lithium metal represents the ultimate high-energy-density battery anode material, its use is limited by dendrite formation and associated safety risks, motivating studies of the solid–electrolyte interphase layer that forms on the lithium, which is key in controlling lithium metal deposition. Dynamic nuclear polarisation enhanced NMR can provide important structural information; however, typical exogenous dynamic nuclear polarisation experiments, in which organic radicals are added to the sample, require cryogenic sample cooling and are not selective for the interface between the metal and the solid–electrolyte interphase. Here we instead exploit the conduction electrons of lithium metal to achieve an order of magnitude hyperpolarisation at room temperature. We enhance the ^7^Li, ^1^H and ^19^F NMR spectra of solid–electrolyte interphase species selectively, revealing their chemical nature and spatial distribution. These experiments pave the way for more ambitious room temperature in situ dynamic nuclear polarisation studies of batteries and the selective enhancement of metal–solid interfaces in a wider range of systems.

## Abstract (web summary)

Understanding the solid–electrolyte interphase (SEI) is key to developing safe dendrite-free lithium batteries. Here, by exploiting the electrons in lithium metal to selectively hyperpolarise the NMR signals, the authors reveal the chemistry and spatial distribution of species at the metal–SEI interface.

## Introduction

The development of longer lasting, higher energy density and cheaper rechargeable batteries represents a major technological challenge^1^ in both the shift from gasoline-powered to electric vehicles and the increased use of typically intermittent renewable energy sources^2^. One strategy to increase energy density is to use lithium metal in place of the commercial anode graphite, in a “lithium metal battery” (LMB)^3,4^. All-solid-state batteries (ASSBs), which replace the flammable organic liquid electrolyte of traditional lithium ion batteries (LIBs) with a safer solid alternative, also require a lithium metal anode if they are to compete with the energy density of LIBs^5^. Similarly, lithium air and sulphur batteries generally use a lithium metal anode^6^. A commercially viable, room temperature, rechargeable battery with a lithium metal anode (LMB or ASSB) has yet to be developed, however, due to difficulties related to plating and stripping lithium and the low Coulombic efficiency caused by reaction of lithium metal with the electrolyte upon cycling^1,3,7^. Mossy and dendritic lithium structures form during plating, which can cause short circuits and severe safety concerns. Lithium deposits can also form on the graphite anode of traditional LIBs upon fast charging, particularly at low temperatures^8^.

While lithium deposition at high rates is largely dictated by the mobility of ions in the electrolyte, Li microstructures are still formed at low rates, in processes that are controlled by the nature of the passivating layer that grows on the lithium metal^4,9,10^. This layer, the solid electrolyte interphase (SEI), is formed by degradation of the electrolyte on contact with lithium metal, resulting in a highly heterogeneous mixed organic/inorganic film^11^. Non-uniform Li^+^ transport across the SEI creates instabilities and nucleation points that determine the extent and nature of the lithium microstructure formation^12,13^.

The SEI has been studied by a range of techniques, including X-ray photoelectron spectroscopy (XPS)^14,15^ and time-of-flight secondary-ion mass spectrometry (TOF-SIMS)^16^; however, the nature and distribution of chemical components in the SEI, the Li^+^ mobility within them, and their relationship to dendrite formation, are still relatively unclear. Significant strides have also been made to study the SEI with NMR spectroscopy^17,18^, however NMR can suffer from issues of sensitivity and selectivity.

Dynamic nuclear polarisation (DNP) is a promising approach that exploits the ~10^3^ times greater gyromagnetic ratio of paramagnetic electrons to hyperpolarise nuclear spins and hence increase the signal in NMR experiments, by irradiating the electron spin resonance (ESR) transitions with microwaves^19–21^. The SEI on reduced graphene oxide and silicon anodes has been studied by exogenous DNP^22–24^, whereby a solution of organic radicals is added to the system before cooling to 100 K or below, slowing the electron relaxation times (*T*_1e_) of the radicals so that the ESR transition can be more easily saturated^20,25^. This approach has several limitations: (i) addition of an organic radical solution may alter the nature of SEI and/or introduce impurities to the system; (ii) the organic radicals are not stable over the large voltage window that most batteries operate in; (iii) the organic radicals are external to the SEI so cannot be readily used to probe the buried SEI–metal interface; (iv) exogenous DNP is not selective to the SEI and can also enhance the signals from any impurities in the system; and (v) low temperature experiments cannot readily be coupled with in situ electrochemical cycling or used to study Li^+^ dynamics. Endogenous DNP has also been used to investigate bulk battery anodes doped with paramagnetic metal ions^26^, but this is not immediately applicable to the study of interfacial structures.

**Fig. 1:**
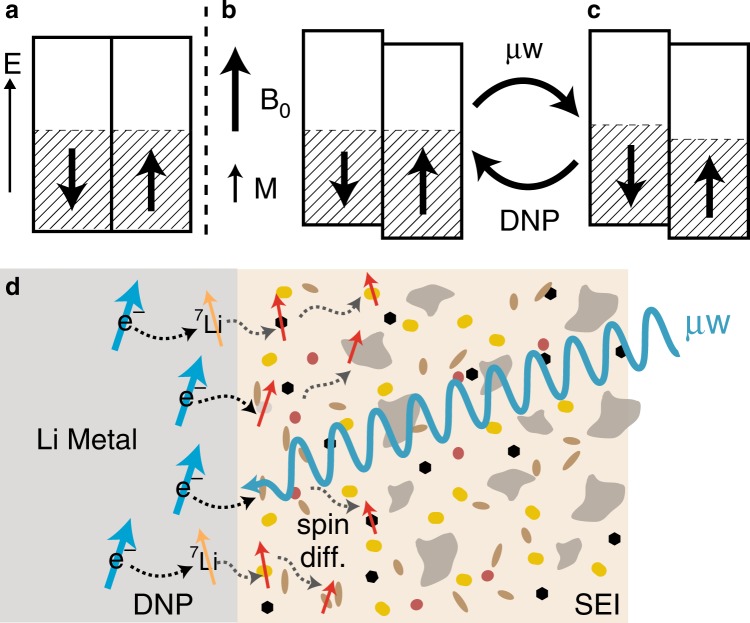
The mechanism of Li metal DNP. **a**–**c** The conduction electron spin-bands of a metal, plotted against energy (*E*): **a** in the absence of a magnetic field; **b** in an applied magnetic field (B_0_), resulting in a Pauli paramagnetic moment (M); and **c** after microwave (μw) irradiation at the CESR frequency. Note that the arrows for each spin-band represent the electron magnetic moment, not the electron spin which is opposite in sign. **d** Schematic of hyperpolarisation of lithium metal on application of microwaves (DNP, black dashed arrows) and subsequent spin diffusion in the heterogenous mixed organic/inorganic SEI (grey dashed arrows).

Intriguingly, the first DNP experiments were performed on lithium metal more than 60 years ago at room temperature^27^ and yet this approach is no longer used. Although the *T*_1e_ of lithium metal is short (~10 ns, depending on the purity^28^, c.f. >10 μs for commonly used organic radicals at 100 K^20^), the relaxation is largely temperature independent^28^, removing the requirement of cryogenic temperatures. Here, instead of localised radicals, the Pauli paramagnetism of the metallic electrons is the source of polarisation. In the absence of an applied magnetic field, the partially filled up and down electron spin-bands of a metal are degenerate (Fig. 1a); in an applied magnetic field the energies of these bands are shifted in opposite direction, but they maintain a common Fermi level—a net unpaired spin density results which augments the applied field (Fig. 1b). This Pauli paramagnetism can be exploited to enhance nuclear magnetisation via the Overhauser effect^29^: on irradiation of the conduction ESR (CESR) transition, the populations of the spin-bands are (partially) equalised (Fig. 1c); the electrons can then cross relax with ^7^Li nuclei in the metal, thereby inducing nuclear hyperpolarisation (see Supplementary Fig. 1). We therefore asked the questions: Could room temperature Li-metal DNP be achieved at high magnetic fields with the much higher power microwave sources now available? And could saturation of the electron spins be used to polarize nearby diamagnetic nuclei? LMBs are well-suited for this study due to the high Li metal surface areas formed on lithium plating.

This paper reports the significant hyperpolarisation of the room temperature ^7^Li NMR signal of cycled lithium metal anodes under magic angle spinning (MAS) by Overhauser DNP. The hyperpolarisation is then harnessed to investigate the interface between Li metal and the SEI (Fig. 1d). Selective enhancement of the SEI is observed in the diamagnetic ^7^Li, ^1^H and ^19^F NMR spectra, and the relative DNP enhancements allow the proximity of different species to the metal surface to be inferred. Double resonance experiments—^7^Li → ^1^H cross polarisation (CP), ^7^Li → ^19^F CP, and ^7^Li{^19^F} rotational echo double resonance (REDOR^30^)—identify further components of the SEI such as polymeric organic species and LiF. The chemical composition and structure of the SEI is highly dependent on the electrolyte system used, so a comparison is made between samples prepared using electrolytes with and without the common additive fluoroethylene carbonate (FEC), which has been shown to yield a more uniform lithium deposition and improved electrochemical performance^31–36^. Finally, experiments on static samples are used to evaluate the feasibility of in situ experiments on working batteries.

## Results

### Lithium metal DNP

**Fig. 2:**
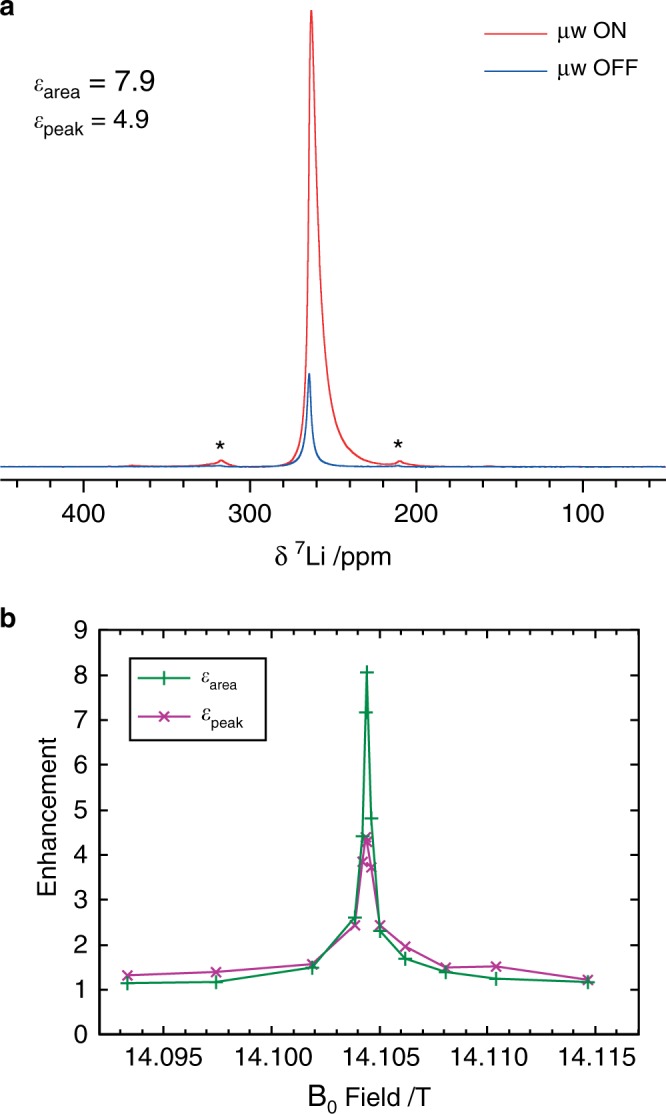
DNP enhancement of Li metal. **a** ^7^Li NMR spectrum of microstructural lithium metal (sample A), with and without 15.6 W of microwave irradiation at 395.29 GHz (μw ON/OFF), recorded at 14.1045 T, 12.5 kHz MAS and a sample temperature of ~300 K, using a Hahn echo pulse sequence, a recycle delay of 0.25 s and 80 scans. Spinning sidebands are marked with an asterisk. **b** The enhancement of the integrated intensity and peak intensity as a function of the B_0_ field (sample B), measured at 100 K.

Figure 2a shows the room temperature (14.1 T) ^7^Li MAS NMR spectrum of microstructural lithium (sample A), with and without 15.6 W of microwave irradiation. The large shift of the Li metal signal (>200 ppm) is due to the Fermi contact interaction with the Pauli paramagnetic moment of the conduction electrons (M, Fig. 1b), a so-called Knight shift^37^. Two clear observations can be made: first, a noticeable enhancement of the lithium metal signal of 7.9 is seen based on the peak area (*ε*_area_); second, microwave irradiation results in a spreading of the NMR signal to lower frequencies, so that the enhancement, based on the peak height is lower (*ε*_peak_ = 4.9). The latter is ascribed to partial saturation of the CESR transition which reduces the moment (Fig. 1c) and hence the Knight shift; the greater the microwave power, the greater the saturation and the lower the observed shift (Supplementary Fig. 2). Early experiments also demonstrated this effect at 0.34 T, where the Knight shift was reduced by ~75% due to the much greater saturation of the CESR transition at the lower field^38^. The distribution of frequencies for the resonance is tentatively ascribed to differences in the degree of CESR saturation in the sample resulting from inhomogeneities of both the sample and the microwave field.

To determine the effect of temperature on the DNP enhancement, the experiment was repeated at 100 K, yielding an enhancement of *ε*_area_ = 7.3 (Supplementary Fig. 3), i.e., of comparable magnitude. A field sweep performed on a second sample (B) confirms the DNP mechanism (Fig. 2b); a single, sharply peaked positive enhancement is observed at the Li CESR frequency, indicating an Overhauser mechanism for which zero-quantum cross-relaxation dominates, given that the gyromagnetic ratio of ^7^Li is positive^20^. The electron *g*-factor corresponding to the peak enhancement is 2.0024 ± 0.0002, compared to 2.0026 from the peak absorbance in the X band ESR spectrum (Supplementary Fig. 4). A narrow sweep at room temperature for a further sample (G) reveals the same sharp feature (Supplementary Fig. 5) at the same field, as expected, since the position of the CESR resonance is temperature independent (Supplementary Fig. 4). Both the field at which the optimal enhancement is achieved (Supplementary Figs. 6 and 7) and the peak absorbance of the ESR spectrum (Supplementary Fig. 8) depend slightly on the applied microwave power, which may be due to partial saturation effects^39^. There is no evidence of a solid effect, which would occur at magnetic fields of ±8.3 mT from the CESR resonance.

Further experiments performed at a lower field strength of 9.4 T with 44 W of gyrotron irradiation on a third sample (C), yielded higher enhancements of *ε*_area_ = 18 at room temperature and *ε*_area_ = 27 at 100 K (Supplementary Fig. 10), the higher enhancements being ascribed to a combination of the higher microwave power achievable, the lower field, and variations between samples. Moreover, even with a lower power klystron source (~2.7 W), enhancements could be achieved at this field on this sample of *ε*_area_ = 7.6 at room temperature and *ε*_area_ = 11 at 100 K (Supplementary Fig. 11), potentially further reducing the technological requirements and cost of running these experiments.

The temperature dependence of the enhancement appears to depend on a number of factors including the magnetic field (see Supplementary Fig. 9), and will also be affected by the temperature dependence of the *T*_1n_ and *T*_1e_ relaxation constants, and hence by the presence of paramagnetic impurities in the sample. For example, the greater enhancement sometimes seen at lower temperatures is at least in part due to differences in the ^7^Li spin lattice relaxation times (*T*_1_) of the metal, which was measured as 0.36 s at 100 K compared to 0.14 s at room temperature (for sample G, Supplementary Table 1). These phenomena are currently under further investigation, but qualitatively similar enhancements are nevertheless achieved between 100 K and room temperature.

### The metal–SEI interface

**Fig. 3:**
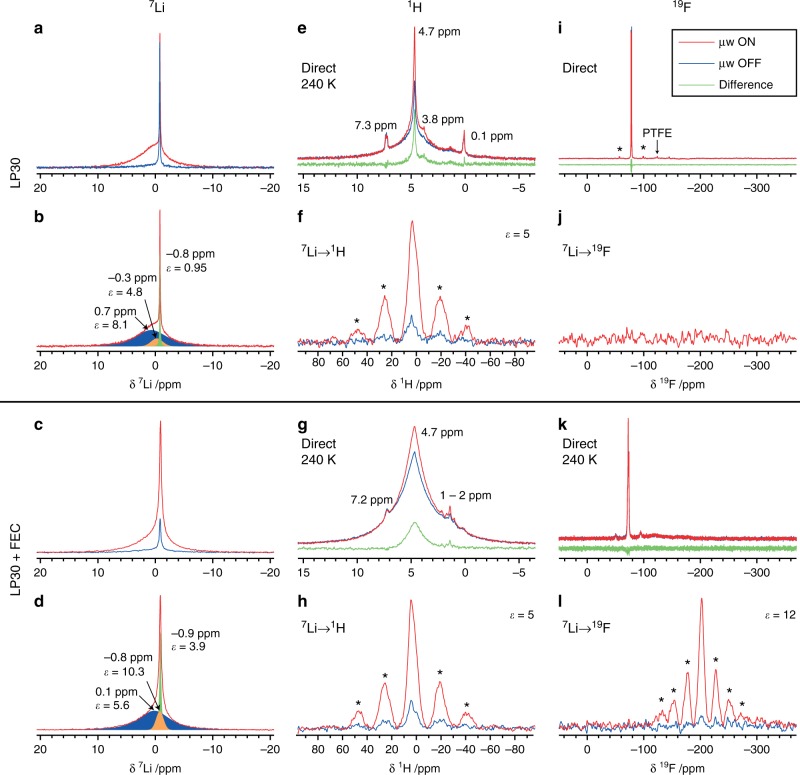
Selective enhancement of the SEI by Li metal DNP. Diamagnetic ^7^Li (**a**–**d**), ^1^H (**e**–**h**), and ^19^F (**i**–**l**) NMR spectra of lithium microstructures produced by cycling with the LP30 (top half, sample D) and LP30 + FEC (bottom half, sample E) electrolytes, recorded with and without 15.6 W of microwave irradiation (μw ON/OFF). (**b**) and (**d**) are deconvolutions of the μw ON spectra in (**a**) and (**c**), respectively; see Supplementary Figs. 15, 16 and Supplementary Table 2. All spectra were recorded at 12.5 kHz MAS, 14.1 T and room temperature, unless otherwise stated. Spinning sidebands are marked with asterisks. The direct spectra were recorded with a Hahn echo pulse sequence and the CP spectra were recorded with the ^7^Li carrier at 0 ppm and contact times of 1 and 0.1 ms for ^7^Li → ^1^H and ^7^Li → ^19^F respectively. For the direct ^1^H and ^19^F experiments, the difference between the spectra recorded with and without microwave irradiation is also shown. Recycle delays and experimental times are given in Supplementary Table 5.

Having demonstrated Overhauser DNP enhancement of the lithium metal signal at high magnetic fields, experiments were then performed to determine if this hyperpolarisation can be exploited to probe the interface between the SEI and lithium metal. Two representative samples were chosen, one prepared with a standard electrolyte LP30, and the second with a 1:10 volume ratio of the additive FEC in LP30 (samples D and E, Fig. 3). Two further samples prepared with the same electrolytes corroborate the results (samples F and G, Supplementary Fig. 13, Supplementary Tables 3 and 4). The metallic ^7^Li signal is again enhanced for all samples (Supplementary Fig. 12), although there is a spread of enhancements (*ε*_area_ = 6–13) that does not correlate with the electrolyte used and is ascribed to factors such as variations in the *T*_1e_ and the surface-to-volume ratio. Importantly, there is still sufficient hyperpolarisation in all cases to investigate the SEI.

Firstly, microwave irradiation of the Li CESR resonance produces clear enhancements in the ^7^Li diamagnetic signals from the SEI that surprisingly are of the same order as that seen for the metal itself (Fig. 3a–d); these must correspond to lithium species within the SEI, such as Li_2_CO_3_ and LiOH^40–42^. The sharp component in the LP30 ^7^Li spectrum at −0.8 ppm is not enhanced and is therefore ascribed to Li^+^ in the residual electrolyte, as the electrodes were not rinsed after disassembly.

The sample formed with the LP30 + FEC electrolyte also exhibits a signal at −0.9 ppm, but it is broader than for the LP30 sample and is enhanced by microwave irradiation (Fig. 3c, d). This suggests that there is residual electrolyte closer to the metal surface, perhaps due to a thinner SEI, or that there is more effective spin diffusion through the SEI due to a greater density of ^7^Li spins. A minor signal is seen at −0.8 ppm with a large enhancement of 10.3, indicating that the corresponding species is close to the lithium metal surface; on the basis of the shift it is tentatively assigned to LiF arising from reduction of FEC^35,36^ and decomposition of the LiPF_6_ salt^43,44^. Similar results are observed at 105 K, but with significantly broader resonances (Supplementary Fig. 14).

Irradiation of the CESR transition is also found to enhance the signal of other nearby nuclei, via mechanisms that will be discussed below. This allows the organic compounds that form a key part of the SEI to be identified without rinsing the electrodes to remove the excess electrolyte and salts that can obscure conventional NMR spectra, a process which may lead to the partial dissolution of the SEI and changes in structure and composition^45^. ^1^H NMR spectra (Fig. 3e, g) were recorded with and without microwave irradiation at 240 K, because microwave irradiation at room temperature can cause melting of ethylene carbonate (see Supplementary Note 2). Although the enhancements are not large, the intensity clearly increases on application of microwaves; furthermore, the spectra are not enhanced uniformly, so the signals arising from the SEI can be distinguished from the significant background intensity.

The ^1^H spectra of the LP30 sample (Fig. 3e, deconvolution in Supplementary Fig. 17 and Supplementary Table 6), show the greatest enhancement for the 3.8 ppm signal, which can only be seen with microwave irradiation, and is assigned to poly-ethylene oxide (PEO)-like species formed through the decomposition of EC molecules; these have been shown to be the major species present in the SEI formed on silicon anodes with an LP30 electrolyte^23,45^. The sharp resonance at 4.7 ppm, which exhibits a comparably large enhancement of ~2, is ascribed to more mobile EC molecules trapped within pores in the SEI^16^, and/or small organic lithium carbonates such as lithium ethylene dicarbonate (LEDC) that are sufficiently close to the metal surface to be enhanced. The same sharp signals are not observed for the LP30 + FEC sample, which suggests that the SEI is less porous to the electrolyte and/or that these organics are further from the Li metal in this case. The broad unenhanced resonance centred at ~4.6 ppm is assigned to solid residual EC electrolyte, broadened by significant homonuclear dipolar coupling. The unenhanced signals at 0.1–0.2 and 7.2–7.3 ppm are impurities (most likely grease and an ammonium salt, respectively).

For the ^1^H spectra of the LP30 + FEC sample (Fig. 3e, deconvolution in Supplementary Fig. 18 and Supplementary Table 6), a noticeably broader signal at ~4.7 ppm is observed with an enhancement of 1.8, from more rigid organic polymers in the SEI such as poly-vinylene carbonate (poly-VC), produced by cross-linking of FEC degradation species; notably, no PEO signal is seen. This is consistent with previous studies on chemically reduced FEC^35^ and again with the SEI seen on silicon anodes^23,24^. The other signals at around 1–2 ppm, seen in both samples with minor enhancements, are ascribed to aliphatic groups, such as in lithium butylene dicarbonate (LBDC)-like species, which form from the reaction of more than one EC/FEC molecule at low potentials^45^.

To improve the sensitivity of the ^1^H spectra, ^7^Li → ^1^H CP experiments were performed which exploit the greater enhancement of the ^7^Li. The room temperature ^7^Li → ^1^H CP spectra (Fig. 3f, h) contain broad asymmetric signals for both samples, which are ascribed to polymeric species and organic carbonates coordinated to or nearby lithium ions^23,45^; these broad signals are obscured in the direct ^1^H spectra. The signal for the LP30 sample has a centre of mass of 2.5 ppm, whereas for the LP30 + FEC sample the centre of mass is at a higher shift of 3.0 ppm; this is consistent with the greater proportion of poly-VC species in the latter due to FEC decomposition. Both samples exhibit noticeable enhancements of around *ε* = 5, which is similar to the enhancements of the diamagnetic ^7^Li, and the species could also be reproducibly identified in the second set of samples (Supplementary Fig. 13f, h). The CP predominantly occurs from the diamagnetic lithium rather than the lithium metal nuclei, as demonstrated by performing experiments with ^7^Li carrier frequencies corresponding to either the metal or diamagnetic ^7^Li signals (Supplementary Fig. 19).

^19^F NMR spectra were acquired to improve the assignments of the species found at the metal–lithium interface. Only the signal from the PF_6_^−^ anion (−72 ppm) can be observed in the direct ^19^F spectra for both samples (Fig. 3i, k); furthermore, this resonance is not enhanced upon microwave irradiation, indicating that the species is not close to the lithium metal, which supports the current understanding that the SEI is impermeable to PF_6_^−^^11^. For the FEC sample, the two signals at around −72 ppm (enlarged in Supplementary Fig. 20) are ascribed to PF_6_^−^ species in different coordination environments; no signal from residual FEC is seen, presumably due to it having evaporated. While no LiF signal (−203 ppm) could be observed in the direct ^19^F NMR spectra for either sample, a clear signal was observed in the ^7^Li → ^19^F CP spectrum for the LP30 + FEC sample (Fig. 3l) with a large enhancement of *ε* ≈ 12. This is more than double the enhancement of the broad diamagnetic ^7^Li signal, which implies that LiF is closer to the metal–SEI interface than other lithium species on average. Again, the CP occurs predominantly from the diamagnetic lithium, rather than from the metal surface, as shown by varying the ^7^Li carrier frequency (Supplementary Fig. 21); however, the enhancement still demonstrates proximity to the metal, since only diamagnetic lithium near the metal surface can be hyperpolarised. The presence of LiF for the LP30 + FEC sample is also supported by ^7^Li{^19^F} REDOR experiments, which exhibit dephasing on ^19^F recoupling (Supplementary Fig. 22), although only for a minor component of the signal. There is a significant enhancement of the difference spectrum, *ε*_area_ = 8 (Supplementary Fig. 23) providing further evidence that LiF in the SEI is close to the metal surface. No LiF is seen for the LP30 sample (D) in the ^7^Li → ^19^F CP or ^7^Li{^19^F} REDOR experiments (Fig. 3j and Supplementary Fig. 24), which is consistent with its formation from FEC reduction. LiF can also form without FEC^32^, and a weak LiF signal was observed for the second LP30 sample (F) in the ^7^Li → ^19^F CP spectrum (Supplementary Fig. 13j), but again significantly more LiF is present near the metal surface for the second LP30 + FEC sample (G, Supplementary Fig. 13l).

### Mechanisms of metal and SEI hyperpolarisation

To explore the selectivity of lithium metal DNP to the metal–SEI interface, first the spatial dependence of the lithium metal enhancement should be considered, which depends on a number of factors. As lithium metal is a conductor, electromagnetic radiation is limited to the skin depth^46^; for microwave irradiation at 395 GHz, the skin depth is ~0.2 μm (see Supplementary Note 1). This would suggest that electrons are only saturated at the surface, in theory limiting the extent of enhancement. However, the paramagnetic electrons near the Fermi level also diffuse rapidly with kinetic energies corresponding to the Fermi energy. An electron retains its saturation on average for a time *T*_2e_, during which it diffuses a distance known as the spin depth, given by $\sqrt {2DT_{2{\mathrm{e}}}}$^47^; for lithium metal at room temperature, using the *T*_1e_ extracted from the power saturation of ~30 ns (assuming *T*_1e_ = *T*_2e_, Supplementary Fig. 25) and the electron diffusivity *D* = 21 cm^2^ s^−1^ ^48^, this yields a spin depth of ~11 μm. Comparing this to the average diameter of the lithium dendrites, ~0.5 μm (see Supplementary Figs. 26 and 27), the electron saturation and hence lithium hyperpolarisation are expected to be uniform within the dendrites. The radiofrequency pulses used to record the ^7^Li NMR spectrum at 233 MHz have a skin depth of ~8 μm, so the microstructural lithium metal is uniformly observed.

Although the hyperpolarisation of the lithium metal is not surface selective on the length-scale of microstructural lithium, the enhancement of the metal–SEI interface is still selective since only the SEI, which is in contact with the metal, will be enhanced (Fig. 1d). Hyperpolarisation of diamagnetic ^7^Li in the SEI can occur either directly via the Overhauser DNP mechanism, if the nuclei are sufficiently close to interact with the conduction electrons, or via spin diffusion from the hyperpolarised ^7^Li metal; evidence of the latter can be seen from a two dimensional exchange (EXSY) spectrum (Supplementary Fig. 29), where cross peaks between the Li metal and diamagnetic Li signals are observed with a mixing time of 100 ms, indicating spin diffusion on this timescale. For both mechanisms, polarisation then propagates away from the metal surface through the diamagnetic ^7^Li nuclei in the SEI by spin diffusion and the selectivity to the SEI–metal interface is therefore determined by the spin diffusion rate and the DNP build-up time used. Propagation of polarisation by physical diffusion of Li in the SEI is another possibility; however, since similar enhancements of the diamagnetic signals are observed at room temperature and cryogenic temperatures (Supplementary Fig. 14 and Supplementary Table 2), this mechanism is unlikely to dominate.

For the direct ^1^H experiments, there are also two possible mechanisms via which the hyperpolarisation could occur. If there are ^1^H nuclei close to the metal surface, they could be hyperpolarised directly via the Overhauser DNP effect, and this hyperpolarisation could propagate through spin diffusion if there is a sufficient density of ^1^H spins. Alternatively, hyperpolarisation could, in principle, be induced by cross-relaxation with hyperpolarised diamagnetic ^7^Li via a nuclear Overhauser effect; experiments to test this proposal are underway. In either case, species closer to the metal surface (i.e., in the SEI) will experience a greater enhancement. The comparatively low direct ^1^H enhancements could be due to a low density of ^1^H nuclei in the immediate proximity of the metal surface, as this region is expected to be more inorganic-rich, and/or due to a relatively inefficient hyperpolarisation mechanism.

### Static lithium metal DNP

**Fig. 4:**
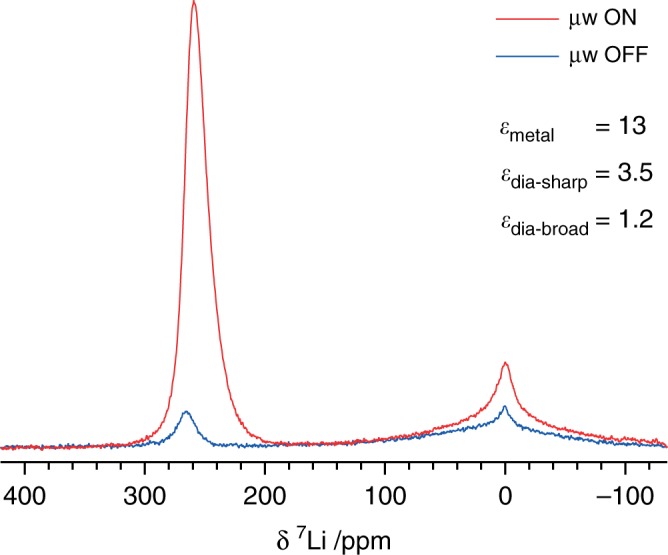
Static DNP-enhanced NMR of microstructural lithium. ^7^Li NMR spectra of sample C, recorded with and without microwave irradiation at 263.7 GHz and 44 W, at 9.4 T and room temperature, using a Hahn echo pulse sequence, a 0.25 s recycle delay and 128 scans. Shown too are the enhancements by area of the different signals.

Finally, although MAS experiments offer the best chemical resolution, it is extremely challenging to perform in situ electrochemical experiments under spinning conditions, and such experiments are necessary to study dynamic processes such as SEI formation and degradation in battery systems, as well as to detect transient species^49^. A MAS cell has recently been demonstrated^50^, but cycling was not performed in the NMR magnet and metal electrodes were not used. To test whether the methodologies presented here could be applicable to in situ studies, ^7^Li NMR spectra were recorded under static conditions at 9.4 T, with and without microwave irradiation (Fig. 4). The resonances are broader, but an appreciable enhancement of the metallic signal is still observed (*ε*_area_ = 13, c.f. *ε*_area_ = 18 under MAS, Supplementary Fig. 10a), as well as minor enhancement of the diamagnetic signal, particularly of the sharper component. Experiments to harness this enhanced polarisation to investigate other nuclei are in progress.

## Discussion

High-field room temperature Overhauser DNP of lithium metal has been demonstrated for samples of electrodeposited microstructural lithium metal, with enhancements of around an order of magnitude. The polarisation source is intrinsic to the system, the addition of potentially reactive radicals is not required, and hyperpolarisation is selectively achieved for the species of interest. The enhancement can also be increased at lower fields and with higher microwave powers. Notably, by removing the need for cryogenic temperatures, the technological requirement for performing such DNP experiments is significantly reduced.

The hyperpolarisation of the lithium metal has further been harnessed to selectively investigate the metal–SEI interface via the ^7^Li, ^1^H and ^19^F NMR spectra, specifically in this case to determine the effect of the FEC electrolyte additive. Selective enhancement of the ^1^H NMR spectra distinguishes the organic SEI components, showing that the addition of FEC to the electrolyte reduces the amount of trapped EC in the SEI and promotes the formation of poly-VC species at the expense of PEO-like species. The greater enhancement of the diamagnetic ^7^Li nuclei can be harnessed to increase the polarisation of ^1^H and ^19^F nuclei via CP, which reveals polymeric species and LiF in the SEI, respectively. The latter has a significantly higher concentration with the addition of FEC. Furthermore, the greater enhancement of the LiF ^7^Li → ^19^F CP signal relative to that of the overall diamagnetic ^7^Li signal provides compelling evidence that this inorganic species is closer on average to the lithium metal surface than other Li-containing species in the SEI. While there are many factors that affect the composition of the SEI, selective observation of the SEI via Overhauser DNP as demonstrated here will help to identify the critical factors for the formation of a stable, uniform, SEI which can minimise lithium dendrite growth in LMBs and other battery systems.

Furthermore, although resolution is reduced, preliminary experiments show that appreciable ^7^Li enhancements can still be achieved by Overhauser DNP under static conditions, which suggests that in situ experiments will be possible whereby DNP can be used to observe the species of interest selectively (e.g. dendritic lithium and/or the SEI), in a working (battery) system where the presence of other components such as the electrolyte would typically drown out such signals. This could allow for the study of SEI formation and growth, one of the fundamental open questions regarding the SEI^11^.

The enhancement afforded by Overhauser DNP of lithium metal could also be applied to investigate very small masses of lithium metal, such as the dendrites or deposits that can also form in commercial lithium-ion batteries due to heterogenous local current densities under fast-charging conditions^8^, as well as in lithium-metal solid-state batteries^5^: such dendrites are challenging to study due to their low concentrations, but understanding how and when they form is of great importance in preventing short-circuits. More generally, this technique allows buried/internal metal–diamagnetic solid–solid interfaces to be directly probed to reveal structural and physical information, even though NMR is typically a bulk technique. In principle, the Overhauser mechanism can operate for any metallic material, and the study of metal anodes for next-generation Na, K and Mg batteries could also be readily envisaged, and even graphitic and LiCoO_2_ electrodes, which are metallic for certain degrees of lithiation. Other applications in solid state chemistry and physics to examine buried metallic–diamagnetic interfaces can be readily imagined.

## Methods

As described in more detail in the Supplementary Information (with a full list of samples A–G given in Supplementary Table 7), lithium metal was deposited onto either lithium disks (samples A, D–G) or copper disks (samples B, C) in coin cells with 75 μL of either 1 M LiPF_6_ in ethylene carbonate/dimethyl carbonate 1:1 vol (EC:DMC; Sigma Aldrich, battery grade), referred to as LP30 (samples B, C, D, F), or the same electrolyte with a 1:10 volume ratio of FEC (Sigma Aldrich, anhydrous), referred to as LP30 + FEC (samples A, E, G). After galvanostatic electrodeposition with current densities ranging from 0.033 to 1.25 mA cm^−2^, the coin cells were disassembled in an Ar atmosphere glovebox and the microstructures scraped off gently with a razorblade without rinsing. The sample was then diluted by ~5× by mass with KBr (previously dried at 200 °C for 24 h) to improve microwave penetration and allow the metallic samples to be more easily spun; this does not have a significant effect on the microstructural morphology (see SEM images, Supplementary Fig. 28). Finally, the sample was packed into a 3.2 mm outer (2.2 mm inner) diameter sapphire rotor.

The majority of the DNP NMR experiments were performed at the UK DNP MAS NMR Facility at the University of Nottingham, UK on a 14.1 T AVANCE III HD spectrometer with a 395 GHz gyrotron microwave source. Additional experiments were performed at Bruker Billerica, USA on a 9.4 T AVANCE III HD spectrometer with a 263.7 GHz gyrotron or a klystron operating at 264.6 GHz. In both cases, a 3.2 mm wide-bore probe was used. Details of the NMR experiments are given in the figure captions and the Supplementary Information. Enhancements, ε, are defined as the signal intensity or area with microwave irradiation, divided by that without.

## Supplementary information

Supplementary Information

## Data Availability

The raw and processed NMR data analysed in the current study are available in the Apollo University of Cambridge repository at 10.17863/CAM.46593.
