# Supplementary material for: Selective NMR observation of the SEI–metal interface by dynamic nuclear polarisation from lithium metal
Source: Nat Commun. 2020 May 6;11:2224. doi: 10.1038/s41467-020-16114-x (PMC7203113; doi:10.1038/s41467-020-16114-x)
Supplement: Supplementary file 1 — Supplementary Information [file 41467_2020_16114_MOESM1_ESM.pdf]

Supplementary Information

Selective NMR Observation of the SEI–Metal Interface by Dynamic Nuclear Polarisation from Lithium Metal

Hope et al.

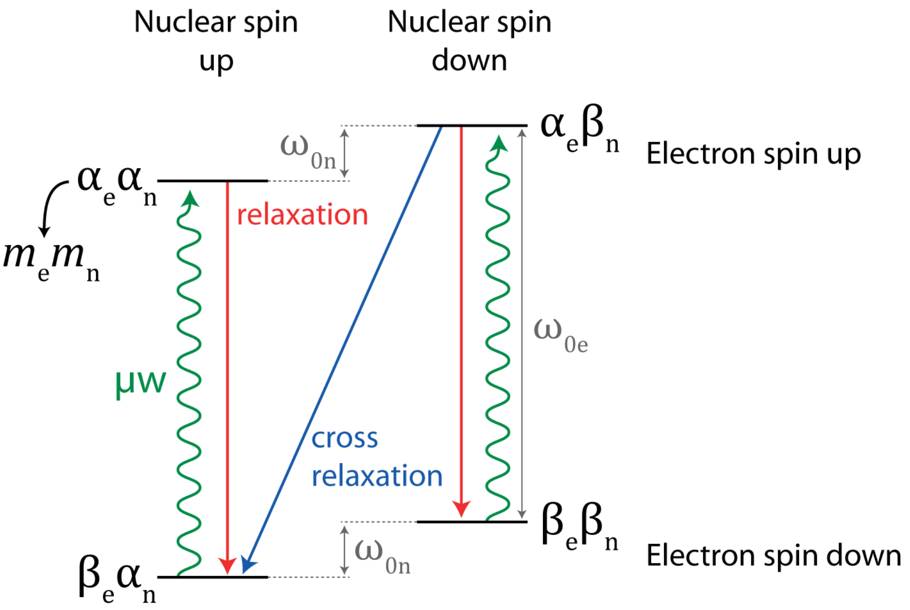

Supplementary Figure 1: **Schematic of the Overhauser Effect.** The four energy levels of a two-spin system, containing one electron spin (m_e) and one nuclear spin (m_n), where α and β represent up- and down- spin states, respectively. The electron states represent a single point in reciprocal space within the band structures shown in Fig. 1b in the main text. The splitting of the electron and nuclear spin levels are denoted by ω_{0e} and ω_{0n}, respectively. On microwave irradiation at the CESR frequency (μw, green wavy lines), electron spins are flipped, populating the upper levels. These electrons then relax to their ground states (red straight lines), but can also cross relax by simultaneously flipping a nuclear spin (blue straight line). Zero quantum cross-relaxation ($\alpha_e\beta_n \rightarrow \beta_e\alpha_n$) dominates because the dominant electron–nuclear interaction is Fermi contact, which is isotropic and cannot drive double quantum cross-relaxation ($\alpha_e\alpha_n \rightarrow \beta_e\beta_n$, not shown).[1–3] Since the cross-relaxation is asymmetric, the net effect is to overpopulate the nuclear spin up state ($m_n = \alpha$), resulting in positive hyperpolarisation of the NMR signal.

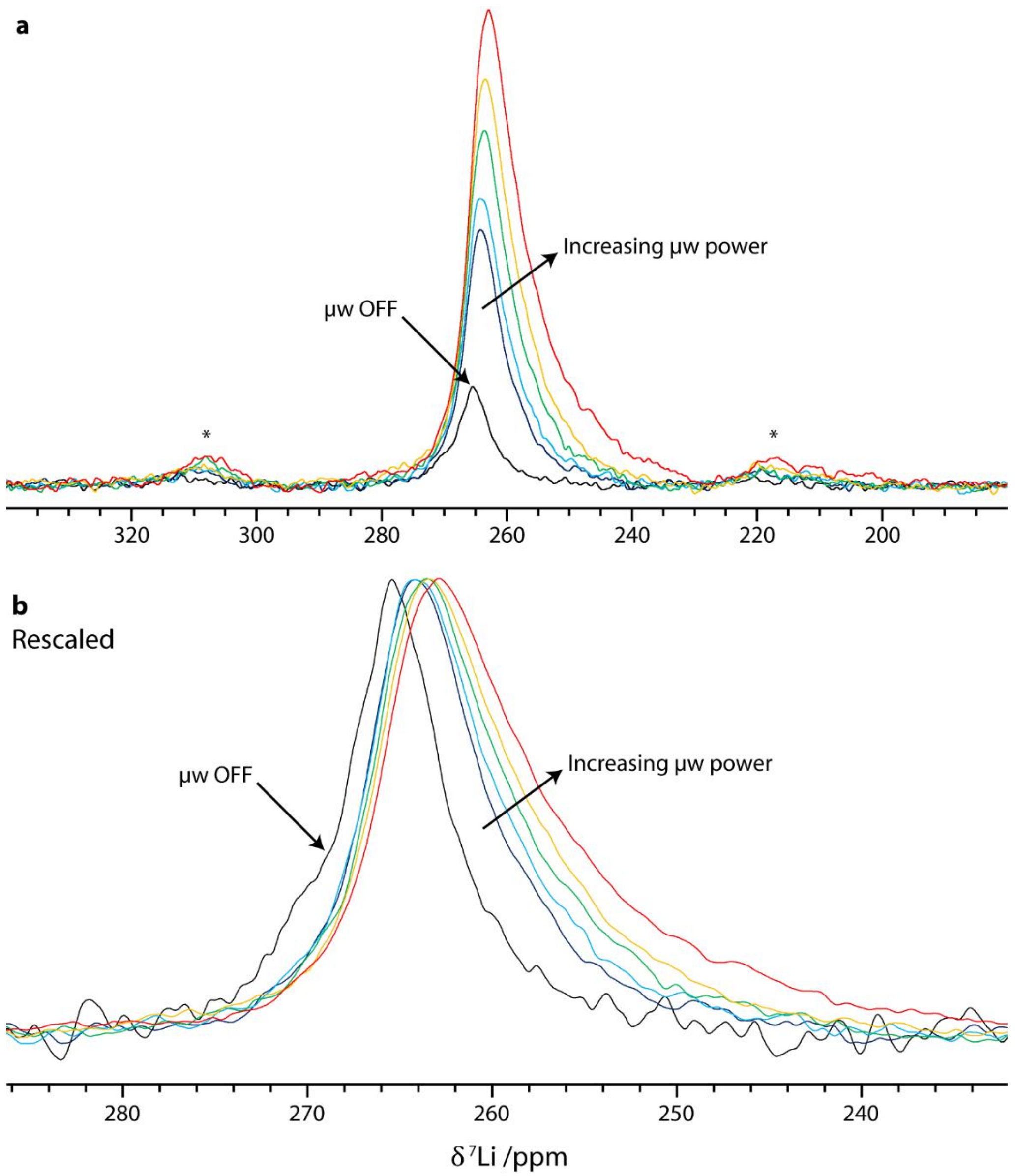

Supplementary Figure 2: **Effect of microwave power on peak position**. ^{7}Li NMR spectrum of microstructural lithium metal (sample B) without microwave irradiation (μw OFF) and as a function of microwave power (7.4, 8.7, 11.0, 13.2 and 15.6 W), recorded at 14.1 T, 10 kHz MAS and a sample temperature of ~100 K, using a Hahn echo pulse sequence and a recycle delay of 0.1 s. Spinning sidebands are marked with an asterisk. The spectra in b) have been rescaled to all have the same intensity, to allow easier comparison of the average chemical shifts.

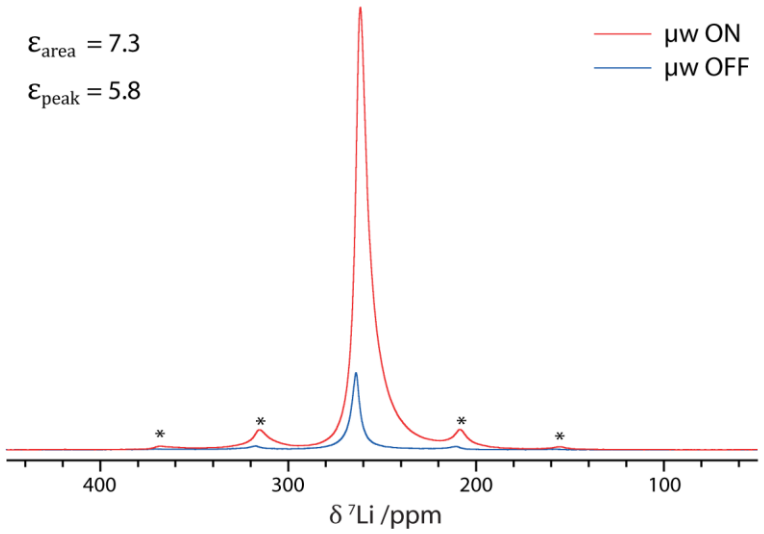

Supplementary Figure 3: **DNP enhancement of Li metal at 100 K.** ^{7}Li NMR spectrum of microstructural lithium metal (sample A), with and without 15.6 W of microwave irradiation at 395.29 GHz (μw ON/OFF), recorded at 14.1045 T, 12.5 kHz MAS and a sample temperature of ~100 K, using a Hahn echo pulse sequence and a recycle delay of 0.25 s. Spinning sidebands are marked with an asterisk.

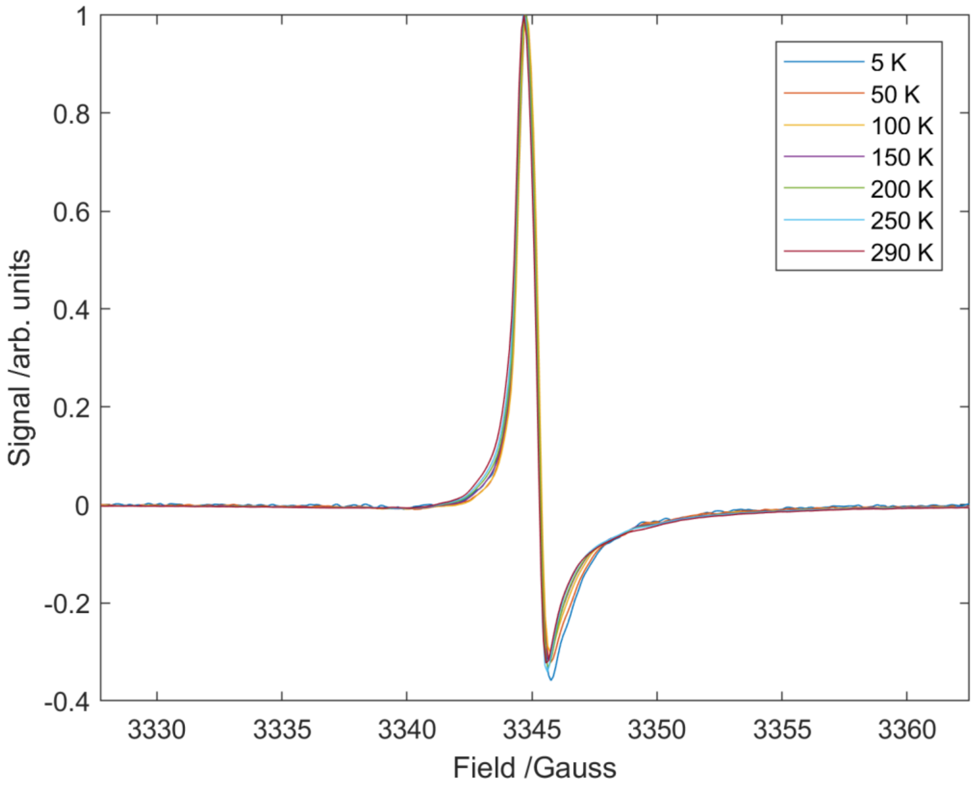

Supplementary Figure 4: **EPR of Li metal as a function of temperature.** The X-band (ν = 9.376393 GHz) EPR spectra of lithium metal as a function of temperature, showing the expected Dysonian lineshape. The microwave frequency changed slightly as a function of temperature, so the magnetic field axes of the low temperature spectra have been rescaled to the 290 K frequency to allow comparison of the spectra, and the intensities have been scaled to the same maximum. The peak absorbance (derivative = 0) occurs at B_0 = 3345.268 G, corresponding to g = 2.0026.

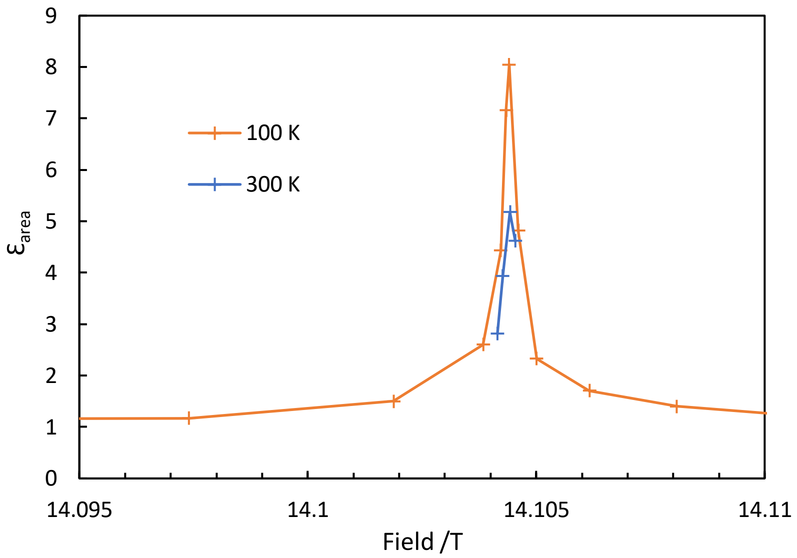

Supplementary Figure 5: **Field sweeps of Li metal DNP at 100 K and 300 K.** The enhancement of the integrated intensity for the metal ^{7}Li NMR signal of microstructural lithium as a function of the B_0 field, measured at 100 K (sample B, Fig. 1b, main text) and 300 K (sample G), with microwave irradiation at 395.29 GHz and 15.6 W, recorded at 12.5 kHz MAS with a recycle delay of 0.25 s.

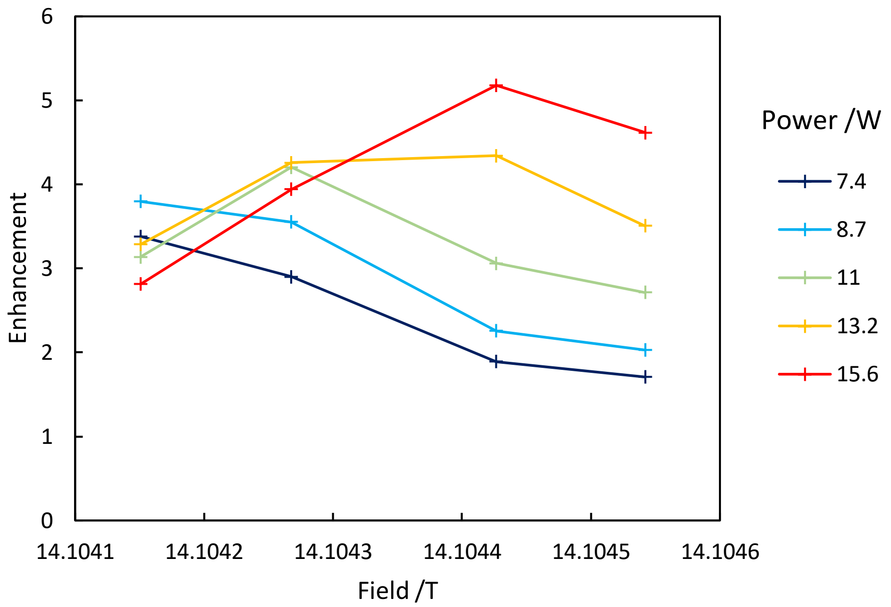

Supplementary Figure 6: **Field sweeps for different microwave powers at ~14.1 T.** The enhancement of the integrated intensity for the metal ^{7}Li NMR signal of microstructural lithium (sample G) as a function of the B_0 field, measured at room temperature, with microwave irradiation at 395.29 GHz and different powers, recorded at 12.5 kHz MAS with a recycle delay of 0.25 s. As the microwave power increases, the field at which maximal enhancement occurs increases.

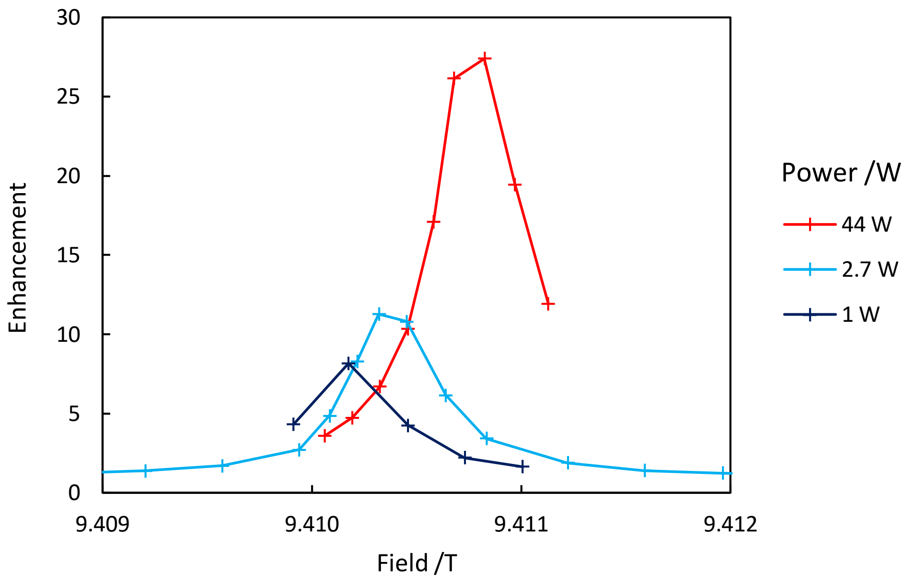

Supplementary Figure 7: **Field sweeps for different microwave powers at ~9.4 T.** The enhancement of the integrated intensity for the metal ^{7}Li NMR signal of microstructural lithium (sample C) as a function of the B_0 field, measured at 100 K with different microwave powers, 8 kHz MAS and a recycle delay of 0.25 s. The 44 W profile was recorded with gyrotron irradiation at 263.7 GHz gyrotron, while the 2.7 and 1.0 W profiles were recorded with a 264.6 GHz klystron. The klystron profiles have been shifted to coincide with the corresponding fields for 263.7 GHz.

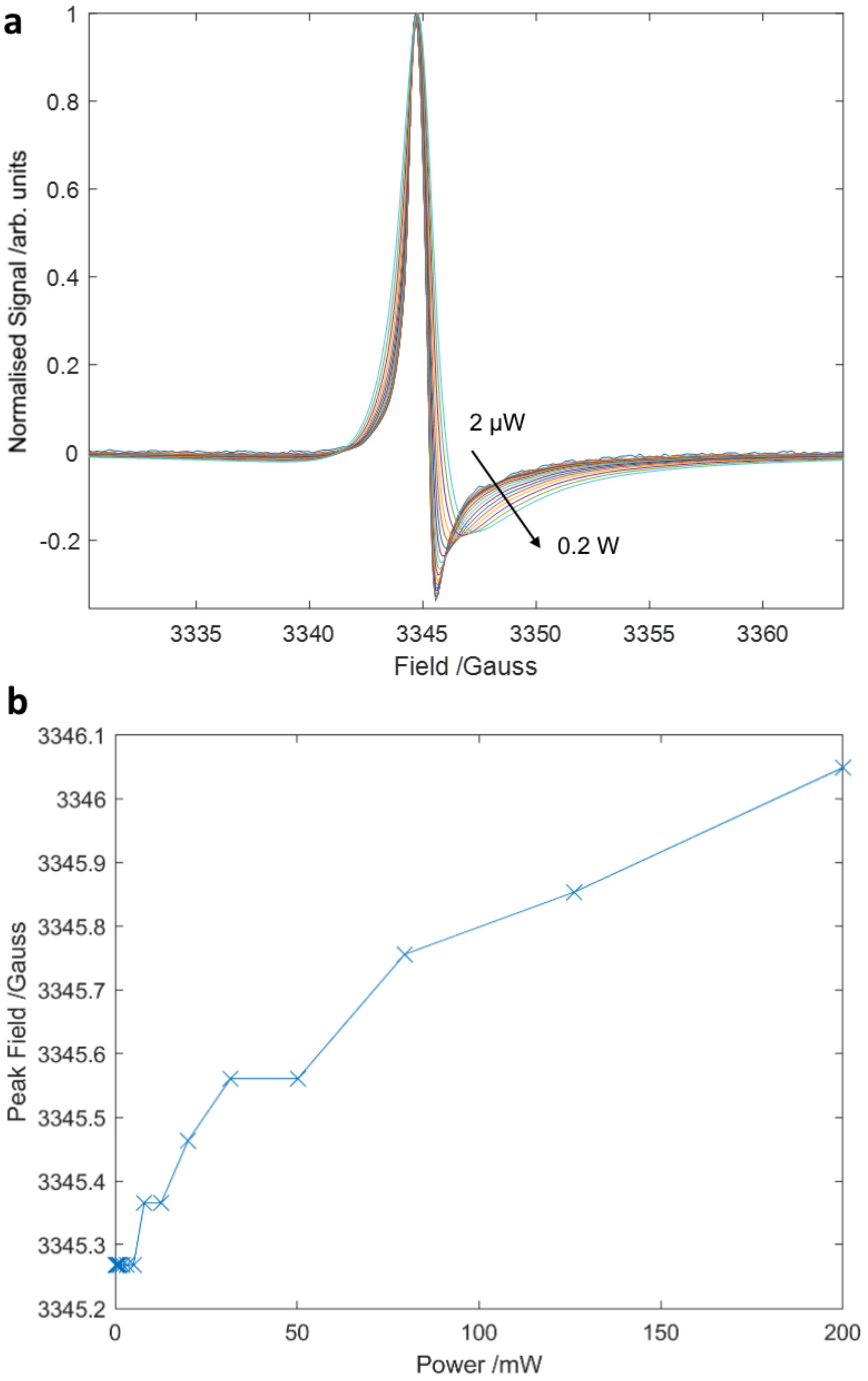

Supplementary Figure 8: **EPR of lithium metal for different microwave powers.** a) The room temperature X-band (ν = 9.376393 GHz) EPR spectra of lithium metal as a function of power, logarithmically spaced between 2 μW and 0.2 W. The intensities been normalised to the same maximum value. b) Magnetic field at peak absorbance (derivative = 0) as a function of microwave power.

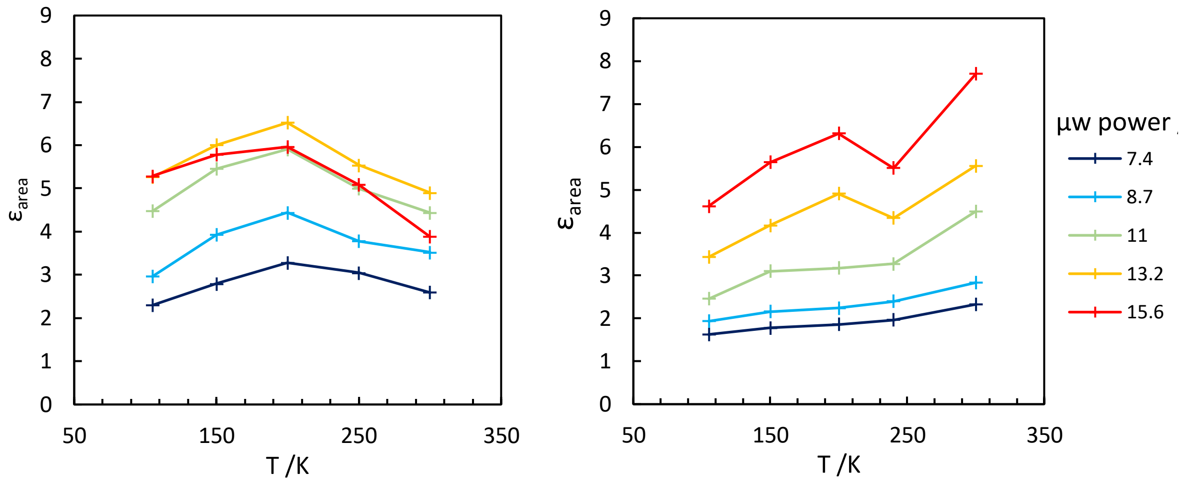

Supplementary Figure 9: **Enhancements as a function of temperature.** Enhancement, by area, of the microstructural ^{7}Li metal signal (sample E) as a function of temperature for different microwave powers. Recorded at 12.5 kHz MAS and 14.1039 T (left) or 14.1042 T (right).

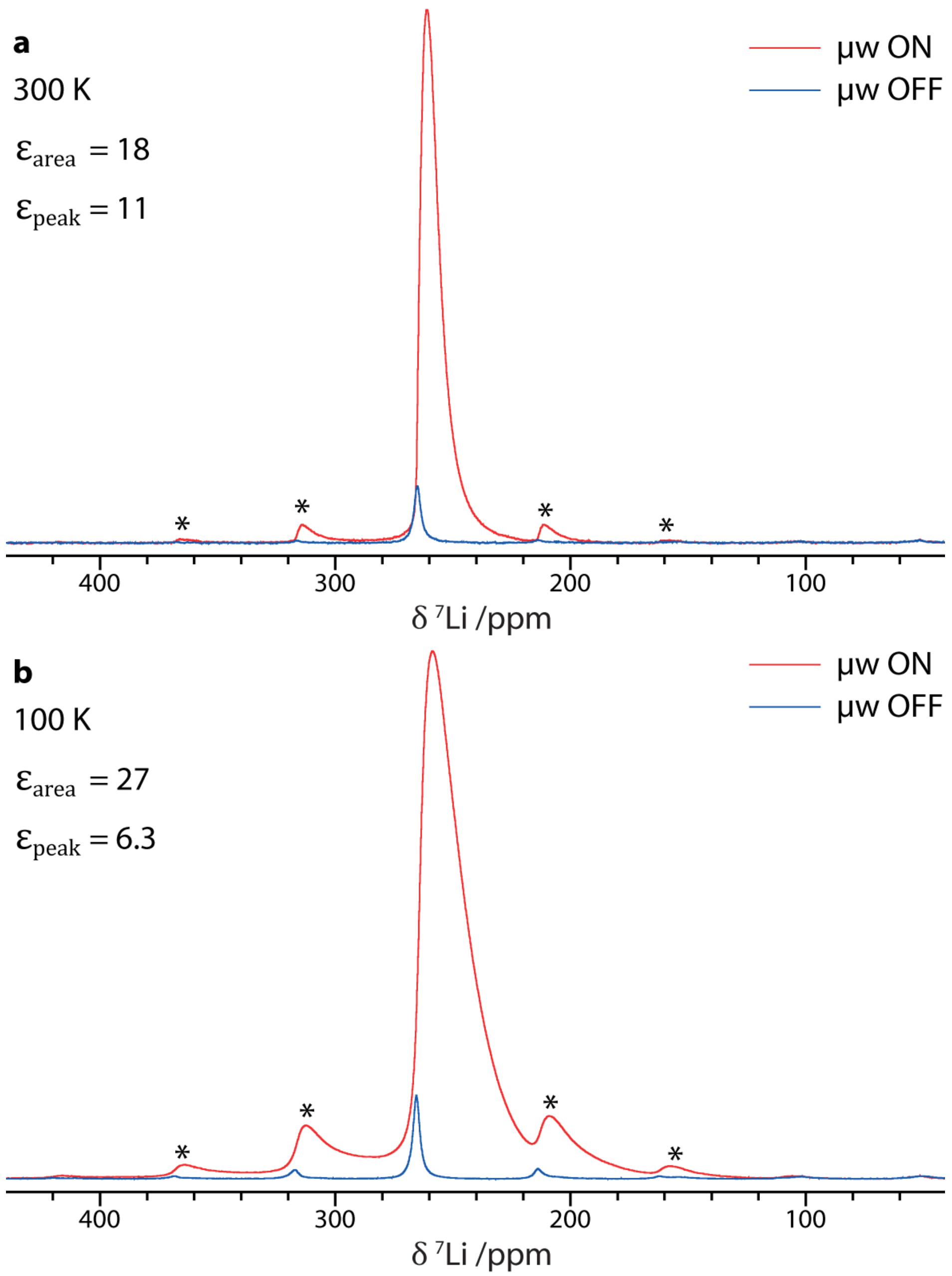

Supplementary Figure 10: **DNP enhancement of Li metal at 9.4 T using a gyrotron.** ^{7}Li NMR spectrum of microstructural lithium metal (sample C), with and without 44 W of gyrotron microwave irradiation at 263.7 GHz (μw ON/OFF), recorded at 9.4 T, 8 kHz MAS and samples temperatures of a) ~100 K and b) ~300 K, using a Hahn echo pulse sequence and a recycle delay of 0.25 s. Spinning sidebands are marked with an asterisk.

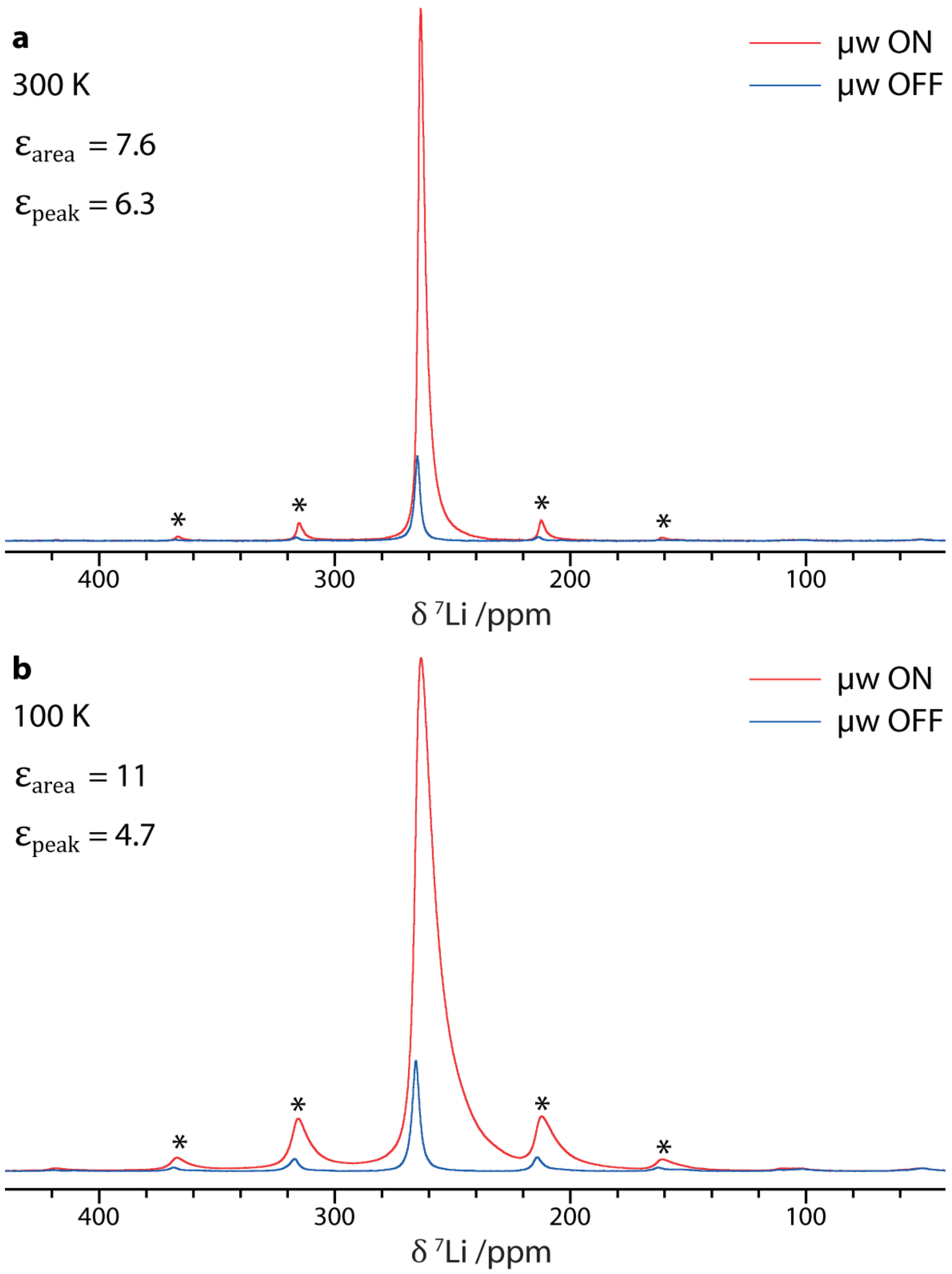

Supplementary Figure 11: **DNP enhancement of Li metal at 9.4 T using a klystron.** ^{7}Li NMR spectrum of microstructural lithium metal (sample C), with and without ~2.7 W of klystron microwave irradiation at 264.6 GHz (µw ON/OFF), recorded at 9.4 T, 8 kHz MAS and samples temperatures of a) ~100 K and b) ~300 K, using a Hahn echo pulse sequence and a recycle delay of 0.25 s. Spinning sidebands are marked with an asterisk.

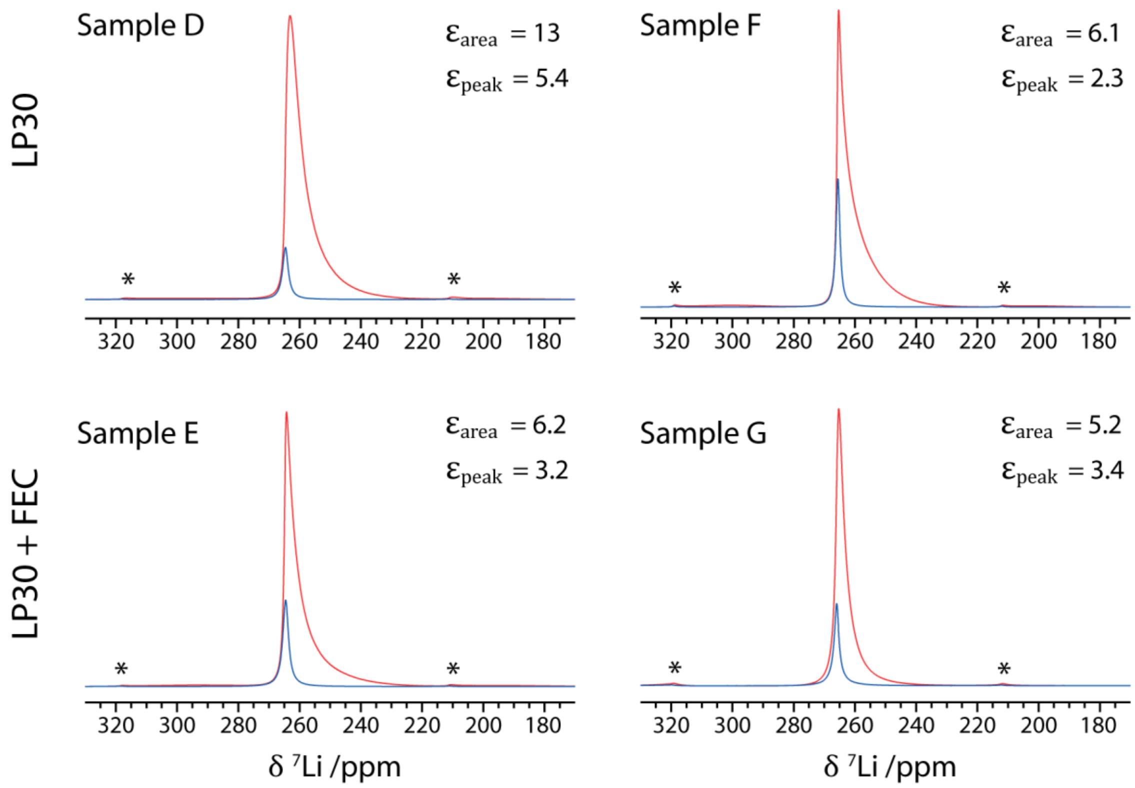

Supplementary Figure 12: **DNP-enhanced spectra of the Li metal signal for samples D – G.** ^{7}Li NMR spectra for both sets of microstructural lithium samples (D – G), deposited using the LP30 and LP30+FEC electrolytes, with and without microwave irradiation at 395.29 GHz (μw ON/OFF; 15.6 W for D, E and 11.0 W for F, G), recorded at 14.1 T, 12.5 kHz MAS and room temperature, using a Hahn echo pulse sequence and a recycle delay of 0.25 s. Spinning sidebands are marked with an asterisk.

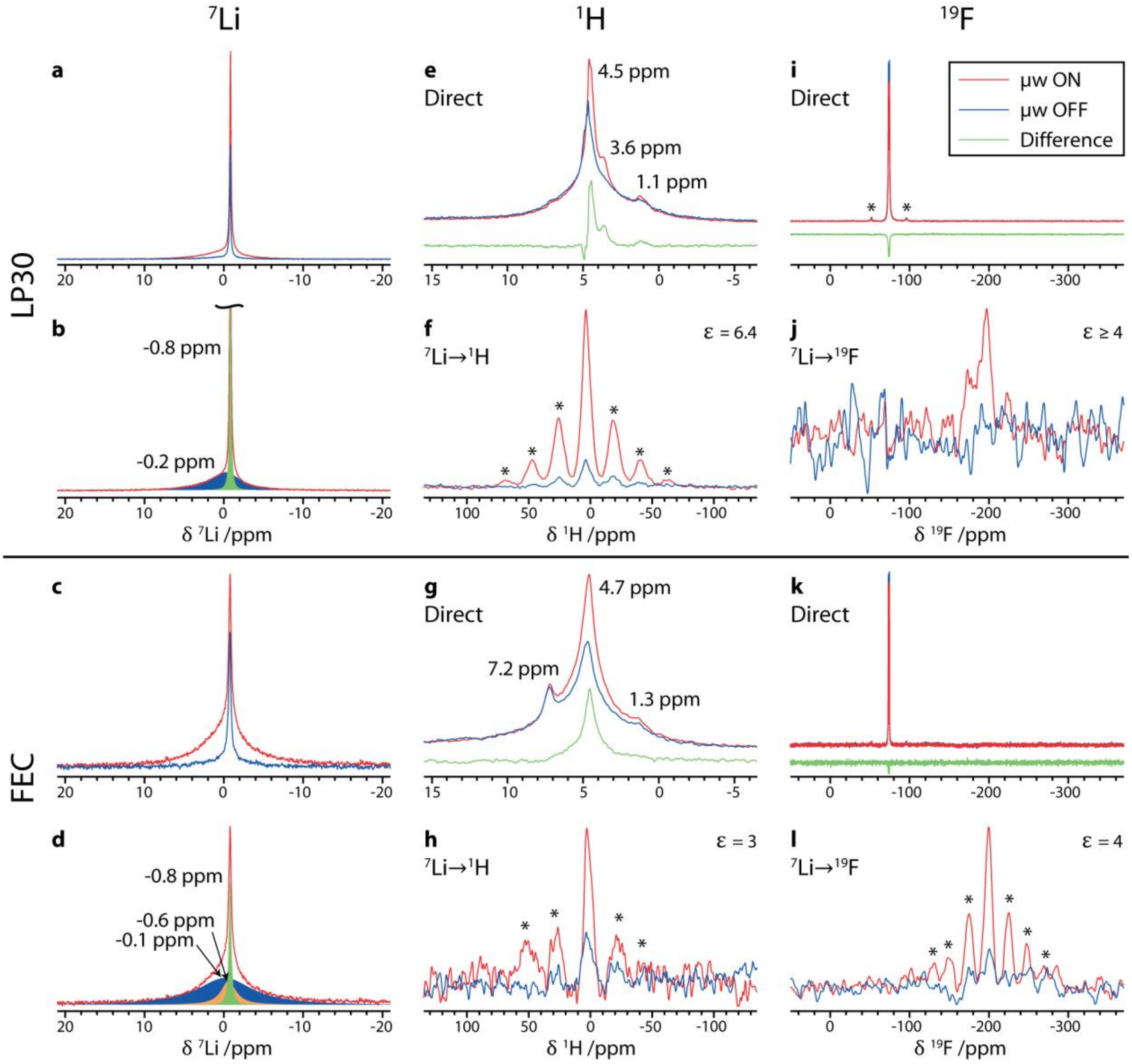

Supplementary Figure 13: **Selective enhancement of the SEI by Li metal DNP for the second set of samples.** Diamagnetic ^{7}Li (a–d), ^{1}H (e–h), and ^{19}F (i–l) NMR spectra of lithium microstructures produced by cycling with the LP30 (sample F, top half) and LP30+FEC (sample G, bottom half) electrolytes, recorded with and without microwave irradiation (μw ON/OFF; 11.0 W for a–i, 15.6 W for j–l). All spectra were recorded at 12.5 kHz MAS, 14.1 T and room temperature, unless otherwise stated; spinning sidebands are marked with asterisks. The direct spectra were recorded with a Hahn echo pulse sequence and the CP spectra were recorded with the ^{7}Li carrier at 0 ppm and contact times of 1 ms and 0.1 ms for $^{7}Li \rightarrow ^{1}H$ and $^{7}Li \rightarrow ^{19}F$ respectively. For the direct ^{1}H and ^{19}F experiments, the difference between the spectra recorded with and without microwave irradiation is also shown. The recycle delays were: 1s (a – d, e, f, h, j, l); 3 s (i, k); or 45 s (g). The deconvolution parameters for (b) and (d) are shown in Supplementary Table 3, and those for (e) and (g) in Supplementary Table 4.

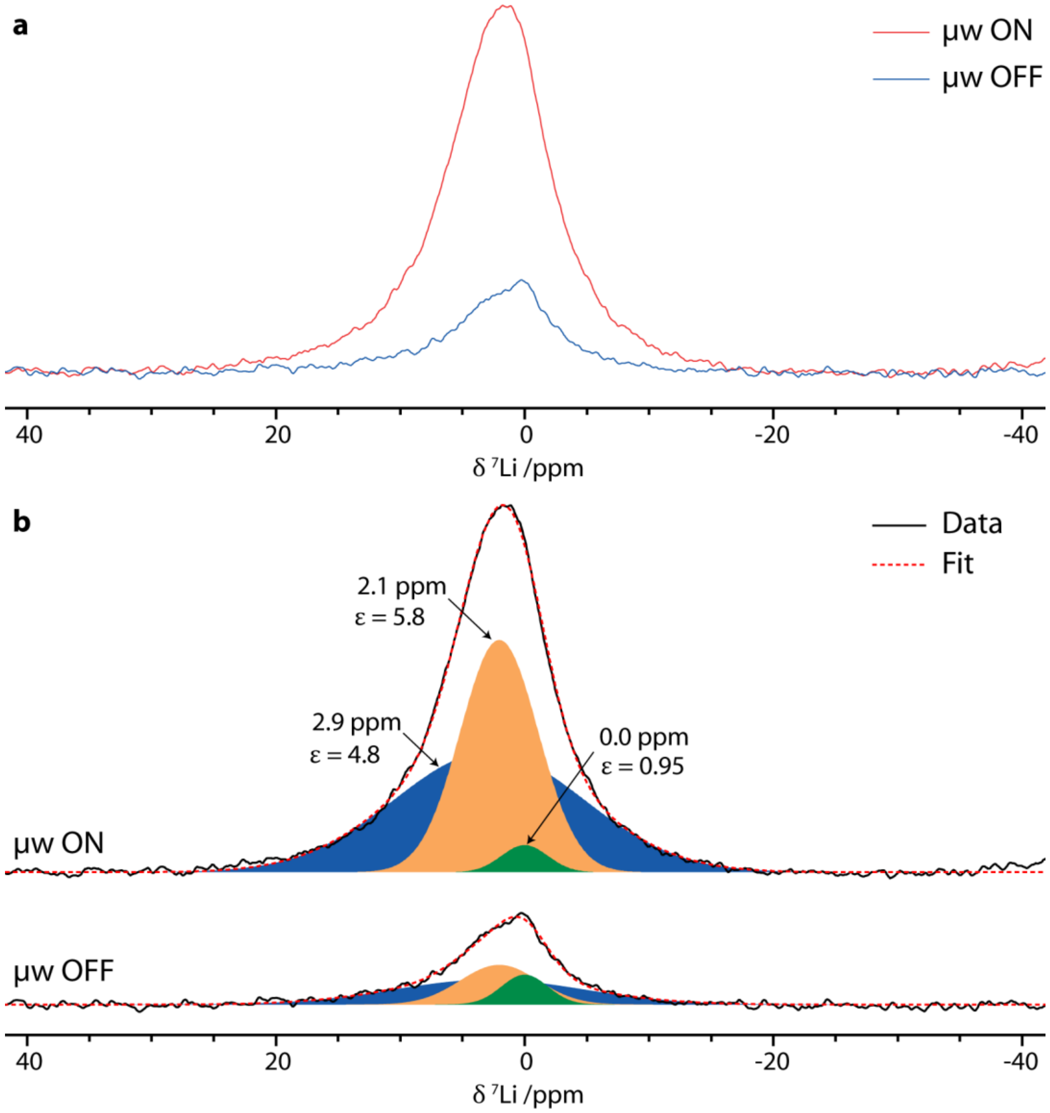

Supplementary Figure 14: **DNP-enhanced spectra of diamagnetic Li at 100 K.** ^{7}Li NMR spectra a) and deconvolutions b) of microstructural lithium deposited with the FEC electrolyte (sample E) with and without microwave irradiation, recorded at 105 K, 14.1 T and 12.5 kHz MAS, with a 1 s recycle delay and a Hahn echo pulse sequence. Similar results are seen as for the room temperature spectra (Main text Fig 3d, Supplementary Fig. 16), but with significantly broader resonances. See Supplementary Table 2.

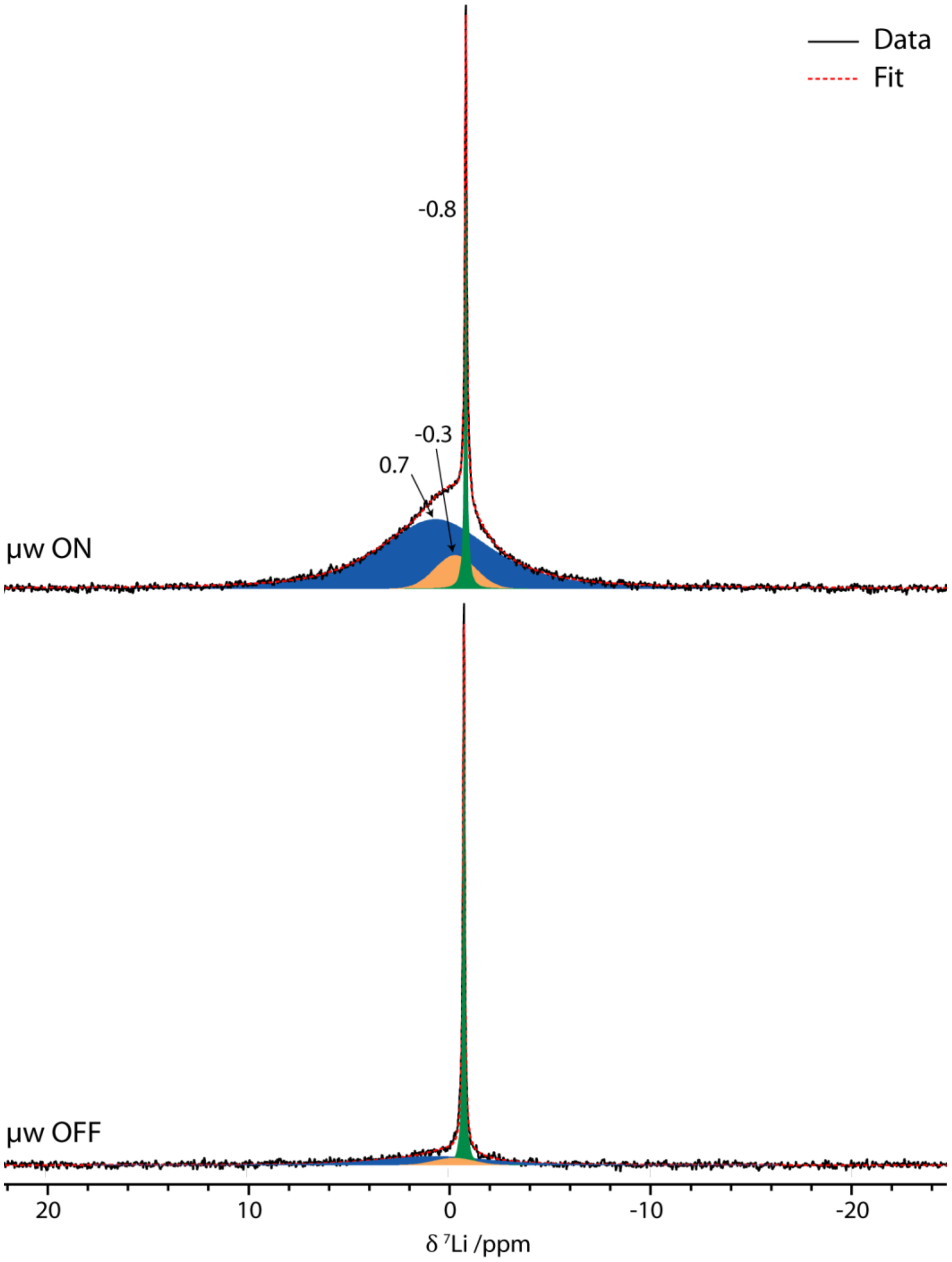

Supplementary Figure 15: **Deconvoluted diamagnetic ^{7}Li NMR spectra for sample D.** ^{7}Li NMR spectra with and without microwave irradiation for microstructural lithium deposited with the LP30 electrolyte (sample D, Fig. 3b, main text), recorded at room temperature, 14.1 T and 12.5 kHz MAS with a 1 s recycle delay and a Hahn echo pulse sequence.

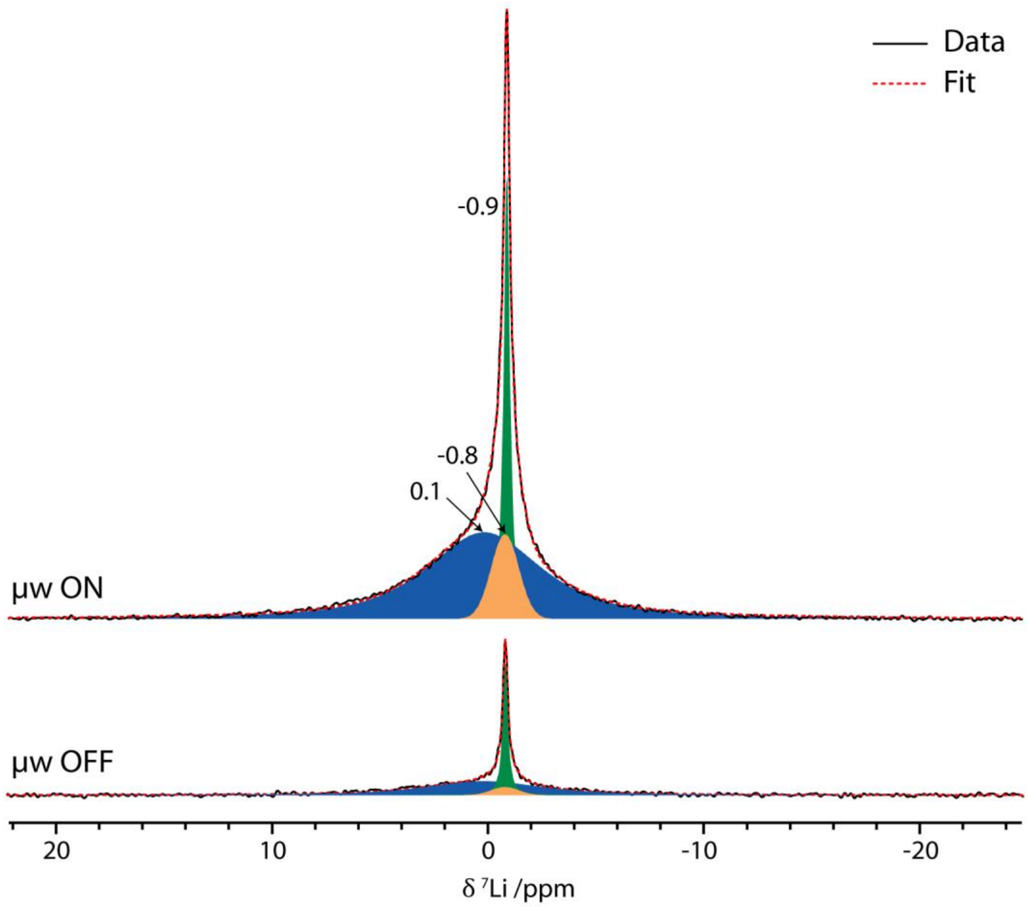

Supplementary Figure 16: **Deconvoluted diamagnetic ^{7}Li NMR spectra for sample E.** ^{7}Li NMR spectra with and without microwave irradiation for microstructural lithium deposited with the FEC containing electrolyte (sample E, Fig. 3d, main text), recorded at room temperature, 14.1 T and 12.5 kHz MAS with a 1 s recycle delay and a Hahn echo pulse sequence.

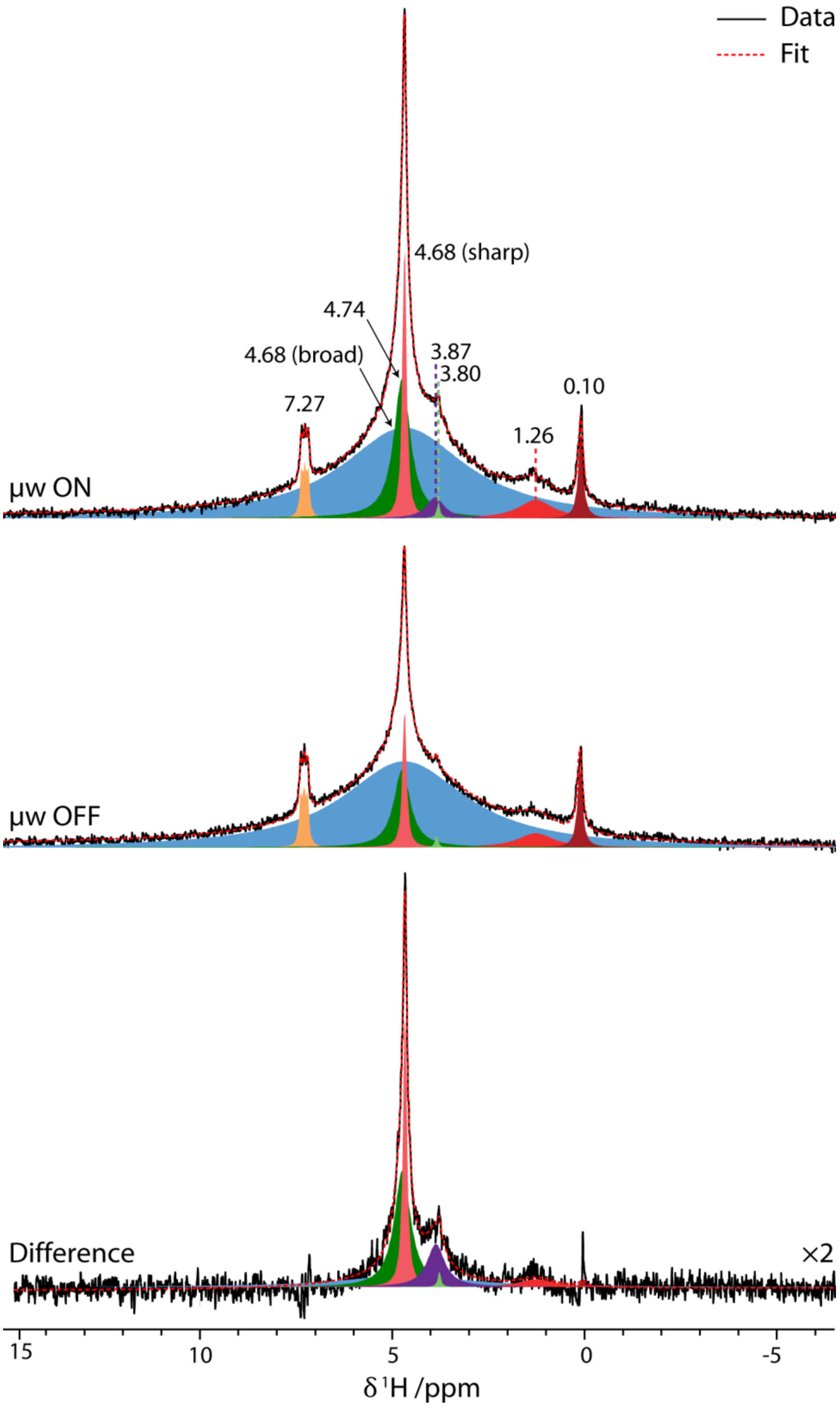

Supplementary Figure 17: **Deconvoluted ^{1}H NMR spectra for sample D.** ^{1}H NMR spectra with and without microwave irradiation and difference spectrum for microstructural lithium deposited with the LP30 electrolyte (sample D, Fig. 3e, main text), recorded at 240 K, 14.1 T and 12.5 kHz MAS with a 10 s recycle delay and a Hahn echo pulse sequence. Deconvolution parameters in Supplementary Table 6.

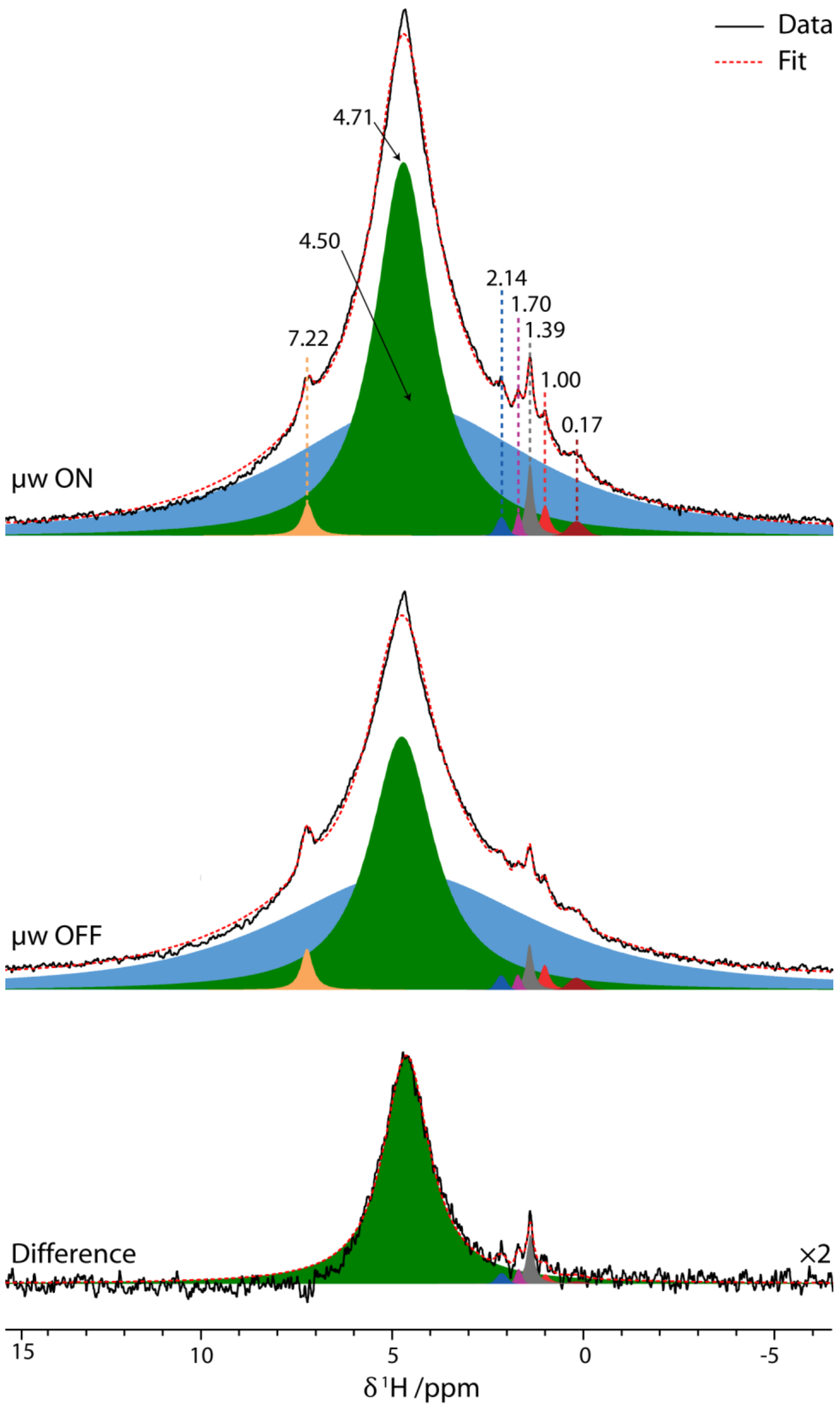

Supplementary Figure 18: **Deconvoluted ^{1}H NMR spectra for sample E.** ^{1}H NMR spectra with and without microwave irradiation and difference spectrum for microstructural lithium deposited with the LP30+FEC electrolyte (sample E, Fig. 3g, main text), recorded at 240 K, 14.1 T and 12.5 kHz MAS with a 10 s recycle delay and a Hahn echo pulse sequence. Deconvolution parameters in Supplementary Table 6.

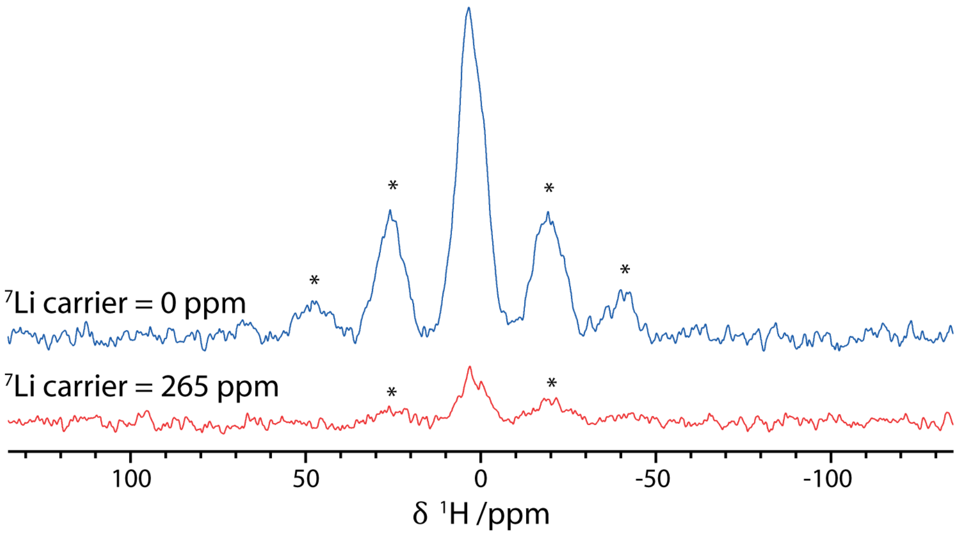

Supplementary Figure 19: **Effect of carrier frequency on ^{7}Li→^{1}H cross polarisation.** ^{7}Li→^{1}H cross polarisation spectra for microstructural lithium produced with the LP30 electrolyte (sample D), with microwave irradiation and two different ^{7}Li carrier frequencies, recorded at 14.1 T and 12.5 kHz MAS using a 1 s recycle delay.

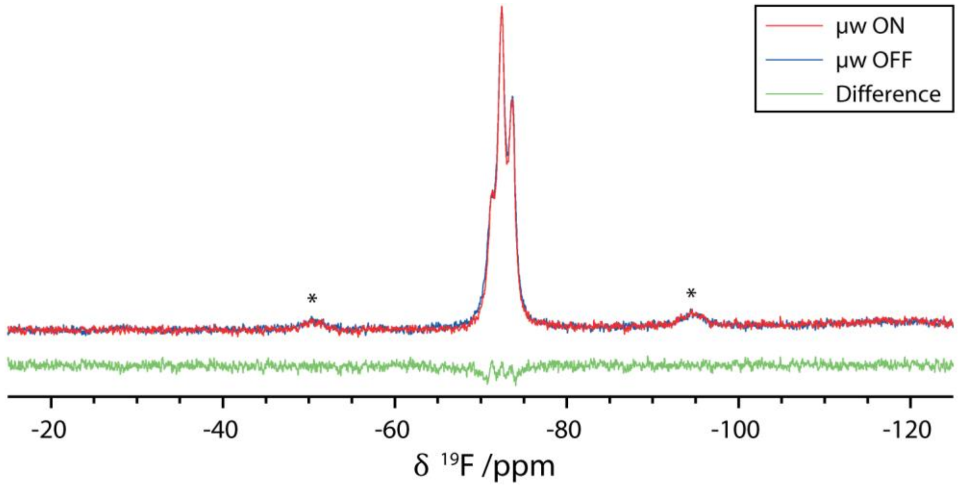

Supplementary Figure 20: **Enlargement of the ^{19}F NMR spectra for sample E.** ^{19}F NMR spectra of microstructural lithium produced with the LP30+FEC electrolyte (sample E), with and without microwave irradiation, recorded at 240 K, 14.1 T and 12.5 kHz MAS using a Hahn echo pulse sequence and a 30 s recycle delay.

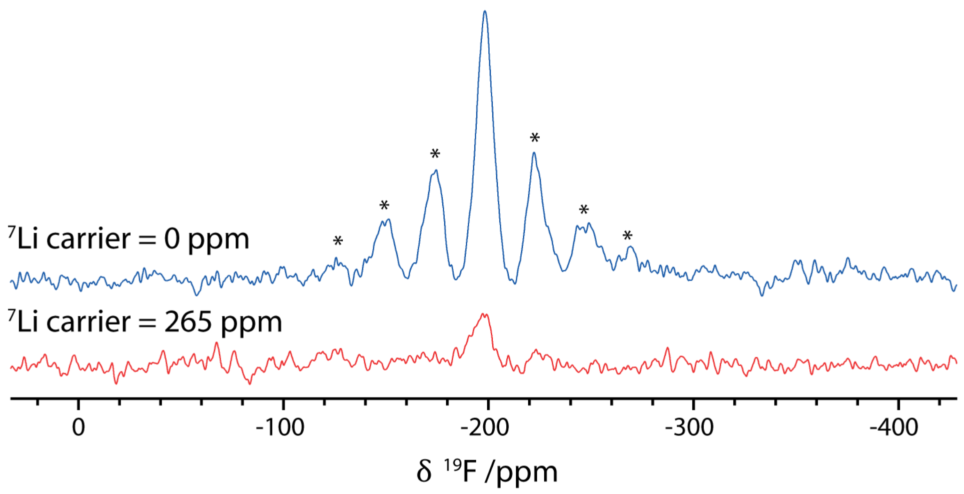

Supplementary Figure 21: **Effect of carrier frequency on ^{7}Li→^{19}F cross polarisation.** ^{7}Li→^{19}F cross polarisation spectra for microstructural lithium produced with the LP30+FEC electrolyte (sample E), with microwave irradiation and two different ^{7}Li carrier frequencies, recorded at 14.1 T and 12.5 kHz MAS using a 1 s recycle delay.

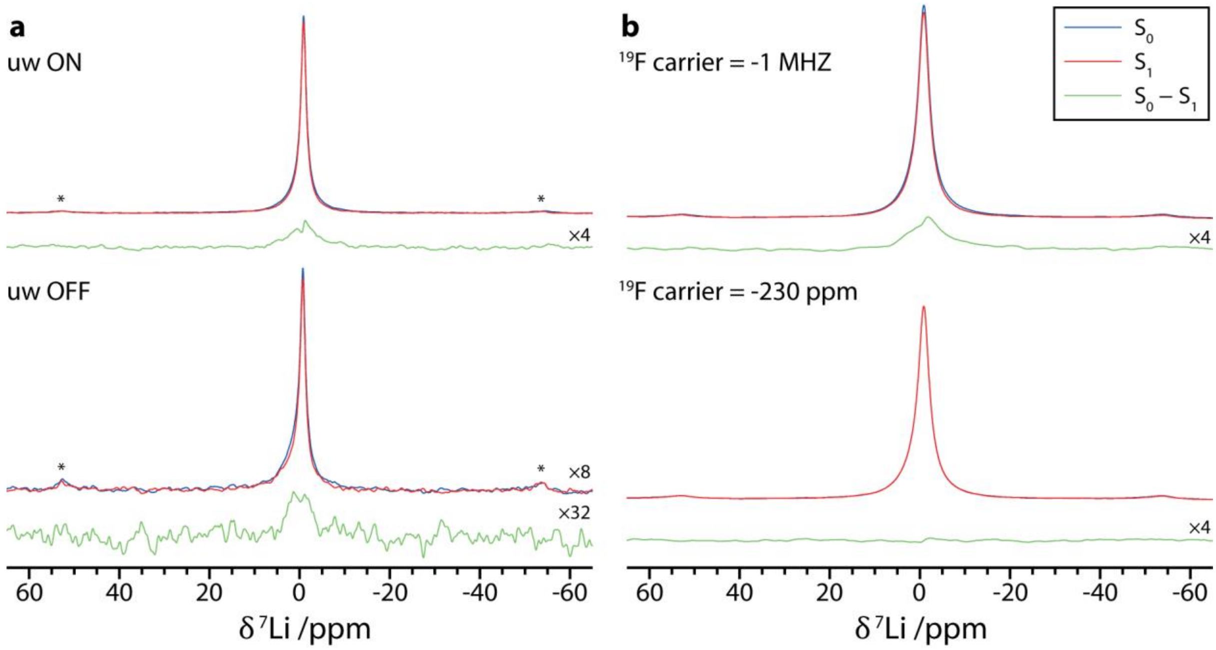

Supplementary Figure 22: **DNP-enhanced ^{7}Li{^{19}F} REDOR for sample E**. ^{7}Li{^{19}F} REDOR spectra of microstructural lithium produced with the LP30+FEC electrolyte (sample E), recorded at 14.1 T and 12.5 kHz MAS, with (S_1) and without (S_0) 5 rotor periods of recoupling. a) Spectra recorded with and without microwave irradiation, using a 10 s recycle delay and 32 scans. b) Spectra recorded with two different ^{19}F carrier frequencies, with microwave irradiation, a 1 s recycle delay and 160 scans. Although the observed dephasing is minor, the fact that it disappears when the ^{19}F carrier is misset indicates that it is a real effect.

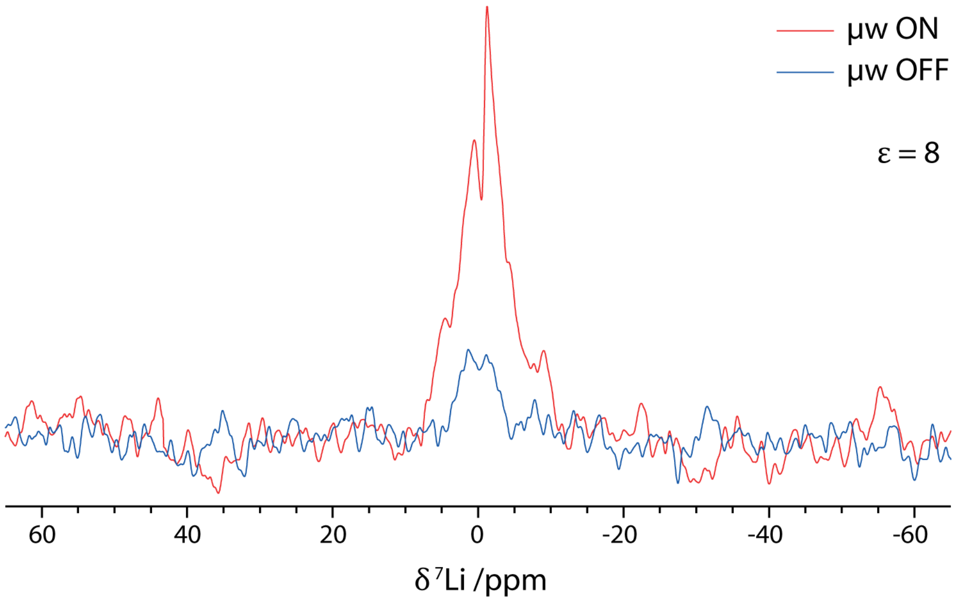

Supplementary Figure 23: **DNP enhancement of the ^{7}Li{^{19}F} REDOR difference spectrum.** Direct comparison of the REDOR difference spectra for microstructural lithium produced with the LP30+FEC electrolyte (sample E), with and without microwave irradiation (see Supplementary Fig. 22).

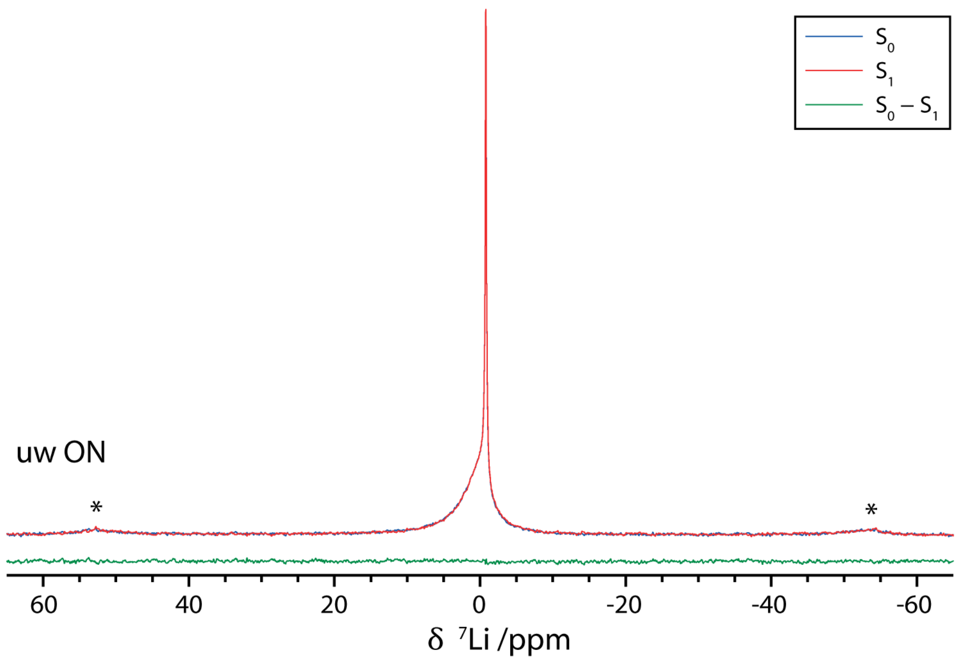

Supplementary Figure 24: **^{7}Li{^{19}F} REDOR for sample D.** ^{7}Li{^{19}F} REDOR spectra of microstructural lithium produced with the LP30 electrolyte (sample D) with microwave irradiation, recorded at 14.1 T and 12.5 kHz MAS, using a 1 s recycle delay, with (S_1) and without (S_0) 5 rotor periods of recoupling, each recorded with 160 scans.

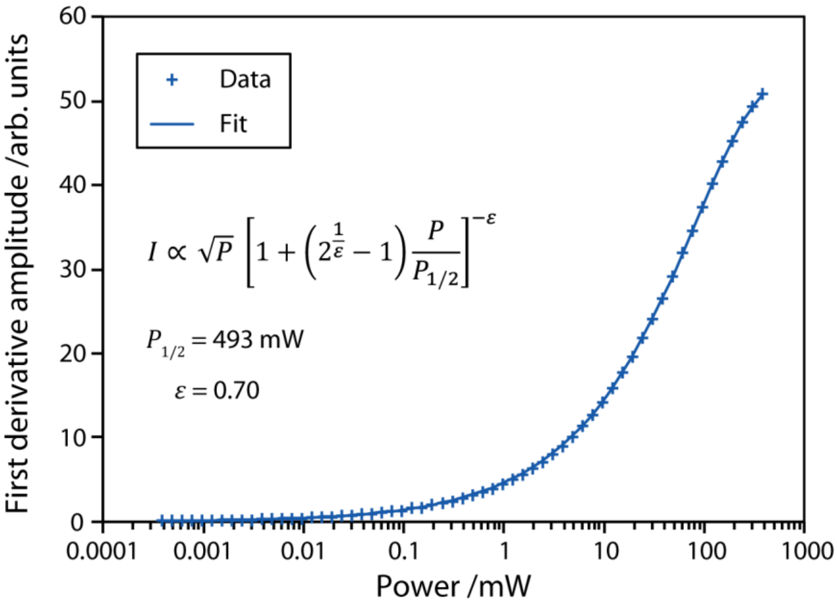

Supplementary Figure 25: $\boldsymbol{T_{1e}}$ **measurement for lithium metal microstructure.** X band ESR saturation curve for microstructural lithium (sample C), showing the amplitude of the first derivative spectrum as a function of the microwave power. The curve was fit to find $P_{1/2}$, the power at half-saturation, using the indicated equation where ε measures the homogeneity of saturation: ε = 0.5 and 1.5 for the inhomogeneous and homogeneous limits, respectively.[4] $P_{1/2}$ is related to the relaxation constants by $P_{1/2} = (2^{2/3} - 1)/(\Lambda^2\gamma_e^2 T_{1e}T_{2e})$, where Λ = 2.2 × 10^{-4} T per $\sqrt{W}$ is the experimentally determined resonator efficiency and γ_e is the gyromagnetic ratio of the electron. Assuming $T_{1e} = T_{2e}$, this yields T_{1e} = 28 ns.

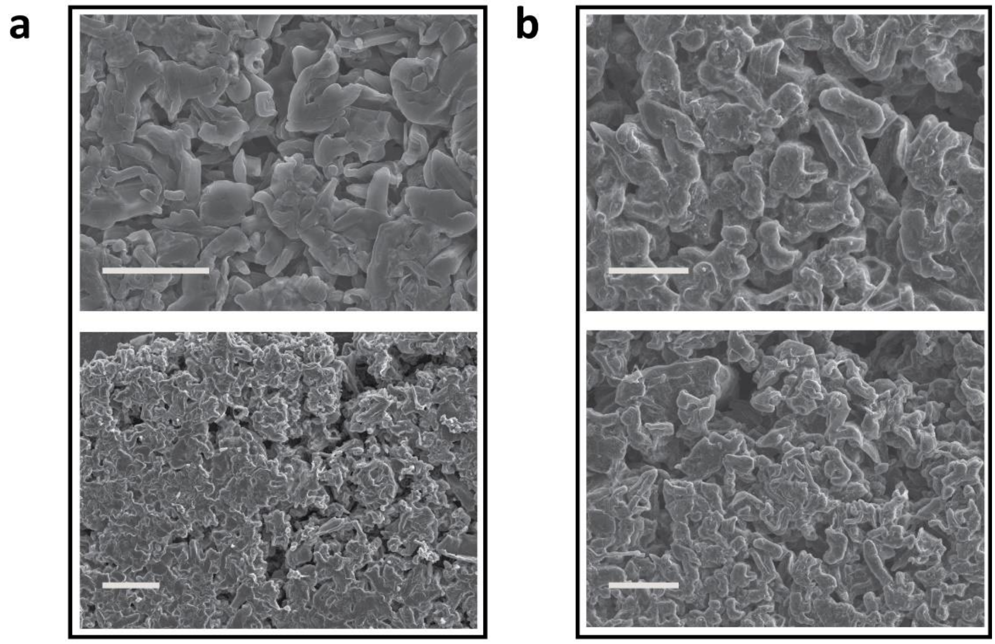

Supplementary Figure 26: **SEM of unscraped lithium microstructure.** SEM images of lithium microstructures deposited on lithium metal disks using a current density of 1.25 mA cm^{-2} and either a) the LP30 electrolyte or b) the LP30+FEC electrolyte. The microstructures were not scraped off the disks. These images show the lithium metal microstructure; the morphology of the SEI is on a nanometre length-scale and cannot be distinguished. The scale bars are 10 μm in the top two images, 20 μm in the bottom two.

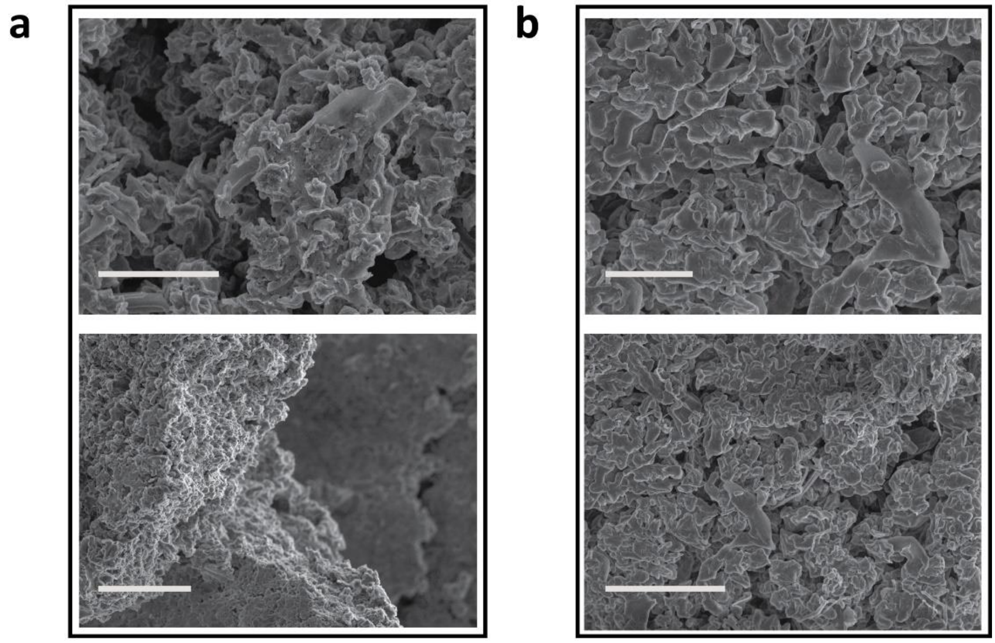

Supplementary Figure 27: **SEM of scraped lithium microstructure.** SEM images of lithium microstructures deposited on lithium metal disks using a current density of 1.25 mA cm^{-2} and either a) the LP30 electrolyte or b) the LP30+FEC electrolyte, after the microstructures had been scraped off the disks. The scale bars are 20 µm in the top two images, 50 µm in the bottom two.

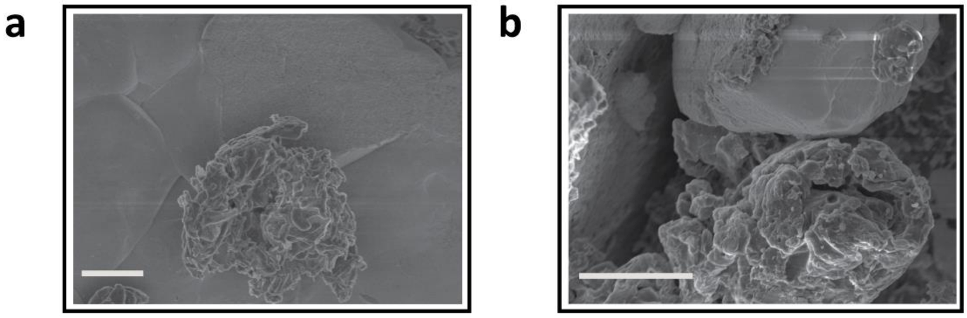

Supplementary Figure 28: **SEM of scraped lithium microstructure, mixed with KBr.** SEM images of lithium microstructures deposited on lithium metal disks using a current density of 1.25 mA cm^{-2} and the LP30+FEC electrolyte, after the microstructures had been scraped off the disks and mixed with KBr with a) light grinding and b) fine grinding. The smooth particles are KBr grains and the porous agglomerates are the lithium microstructure. Although the agglomerates are smaller after grinding, as desired, the microstructure is very similar. The scale bars are 10 μm.

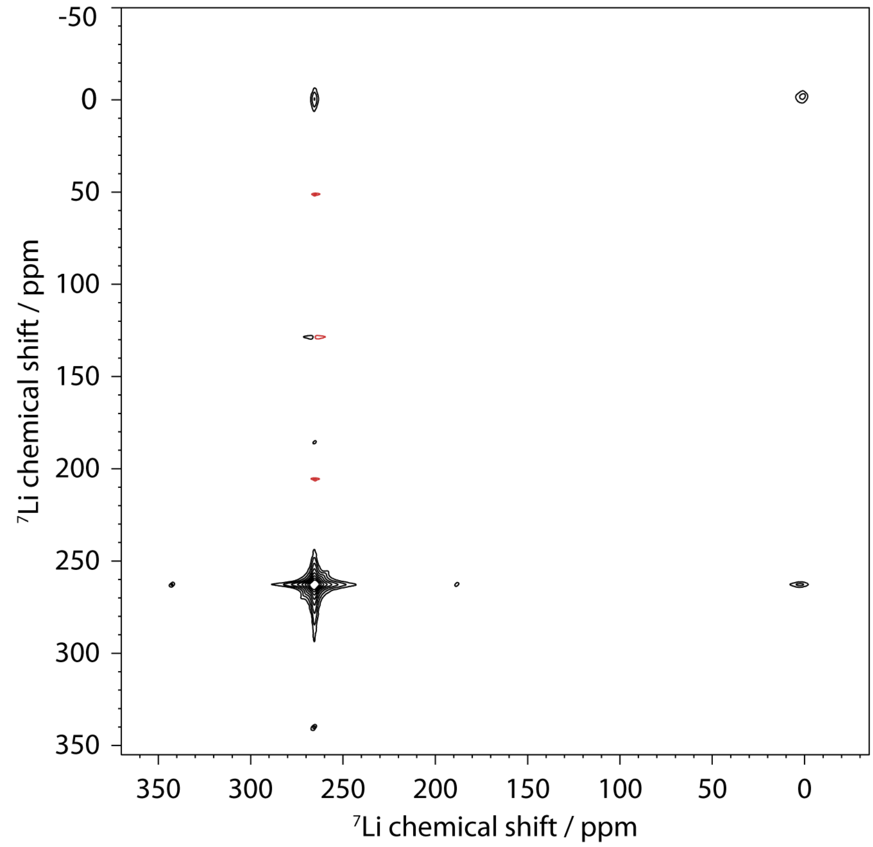

Supplementary Figure 29: **^{7}Li EXSY spectrum of microstructural lithium**. Echo-detected ^{7}Li EXSY spectrum of a microstructural lithium sample prepared in the same way as samples D and F, recorded at 11.7 T and 15 kHz MAS with a mixing time of 100 ms, a recycle delay of 1 s and an rf-field strength of 70 kHz. 425 complex t_1 points were acquired using States quadrature detection, leading to 4.7 ms of acquisition time in the indirect dimension. The cross peaks between the metal (265 ppm) and SEI (0 ppm) signals indicate spin diffusion, since physical exchange involving motion of the lithium between Li metal and the SEI is a process which takes minutes to hours.[5]

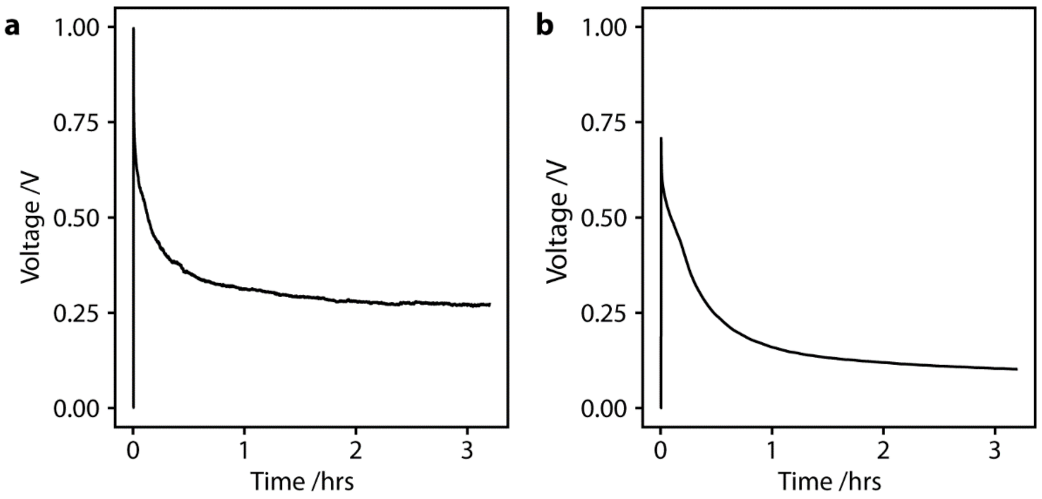

Supplementary Figure 30: **Representative electrochemistry for Li–Li symmetric cells.** a) with the LP30 electrolyte and b) with the LP30 + FEC electrolyte. Shown for samples D and E, respectively, with plating at a constant current of 1.25 mA cm^{-2} for 3.2 hours (4 mAh cm^{-2}).

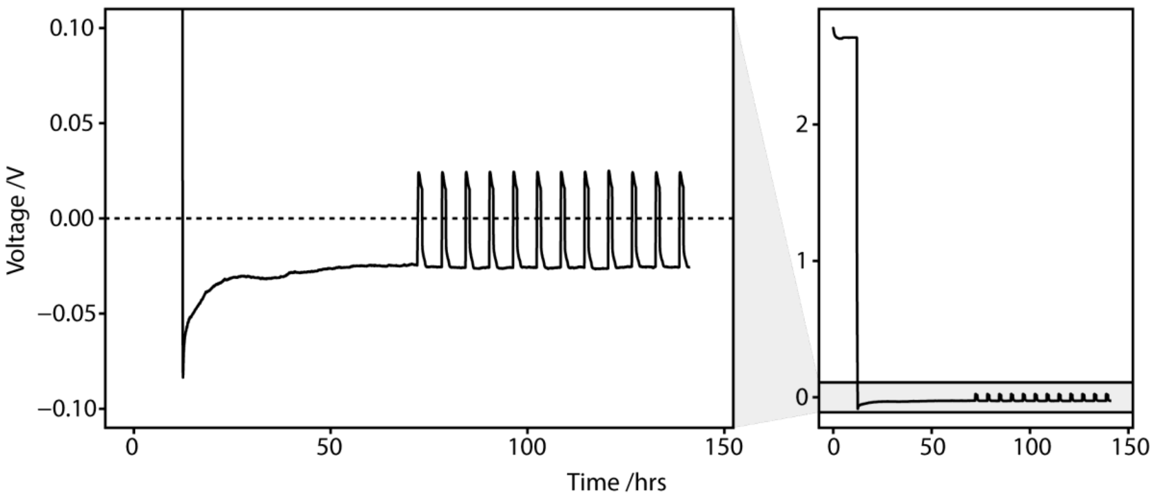

Supplementary Figure 31: **Electrochemistry for sample B**. A Li–Cu cell with the LP30 electrolyte: plating at a constant current of 0.033 mA cm^{-2} for 60 hours (2 mAh cm^{-2}), followed by 12 cycles of stripping for 1 hour and plating for 5 hours, each at 0.033 mA cm^{-2}.

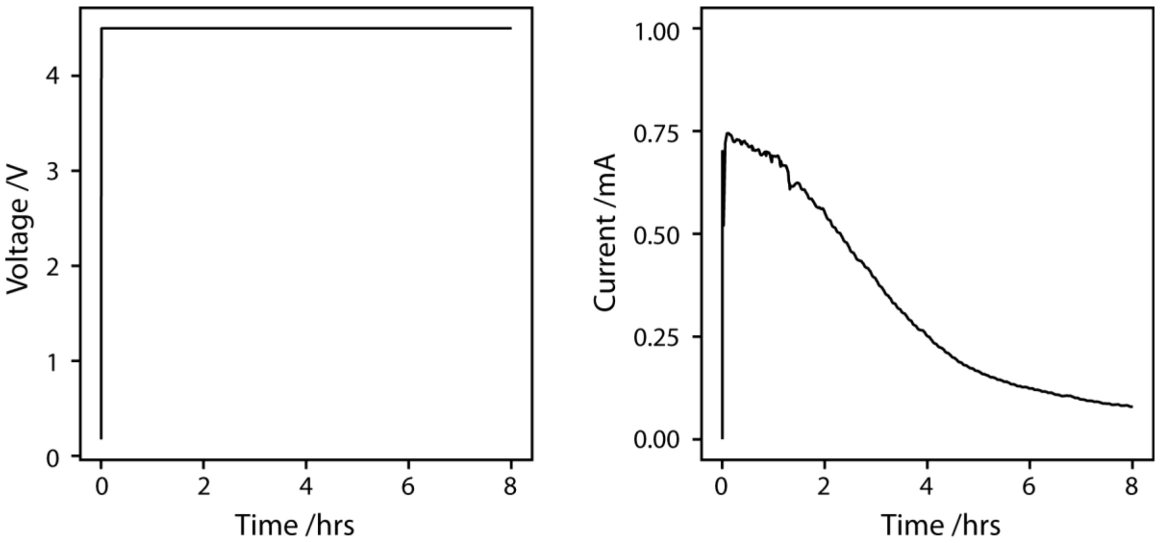

Supplementary Figure 32: **Electrochemistry for sample C**. An NMC811–Cu cell with the LP30 electrolyte. A constant current was applied until the voltage reached 4.5 V, before holding at 4.5 V for 8 hours.

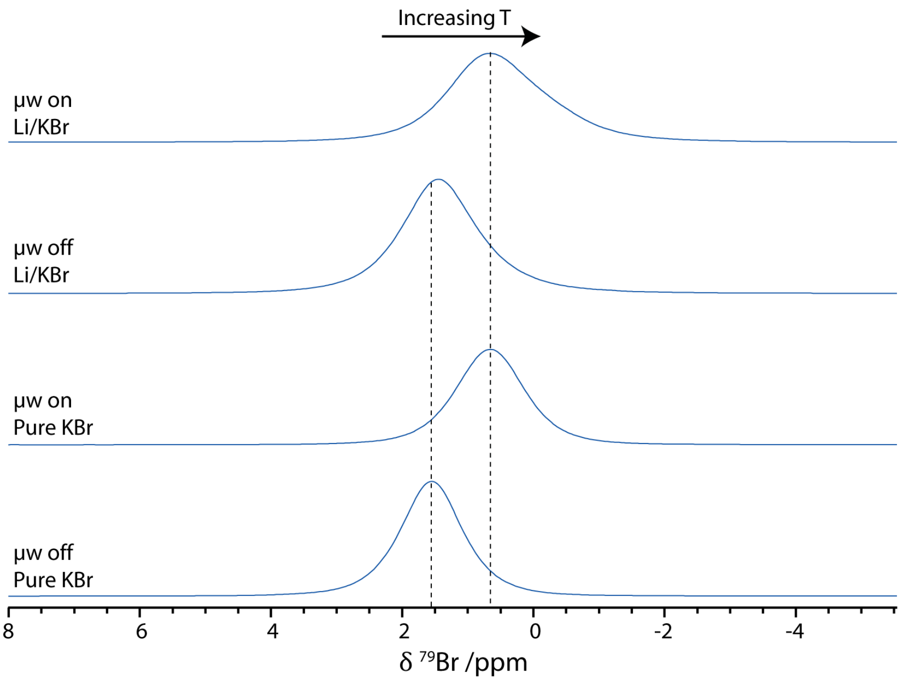

Supplementary Figure 33: ^{79}Br NMR spectra (14.1 T) of a microstructural lithium sample mixed with KBr and a pure sample of KBr, with and without ~15.6 W of microwave irradiation (395 GHz) at room temperature under 12.5 kHz MAS.

Supplementary Table 1: Enhancements and T_1 and T_{DNP} build-up constants, where measured, for the metal ^{7}Li signal for different microstructural lithium samples. T_1 and T_{DNP} were measured with saturation recovery experiments with and without microwave irradiation, respectively.

Sample	Field /T	ν_{MAS} /kHz	µw power /W	T /K	ε_{area}	T_1 /s	T_{DNP} /s
A	14.1	12.5	15.6	100	7.3	–	–
				300	7.9	0.14	0.13
B	14.1	10	15.6	100	8.1	–	0.25
C	9.4	8	2.7	100	11	0.45	0.31
				300	7.6	–	0.13
			44	100	27	–	–
				300	18	–	–
D	14.1	12.5	15.6	300	13	–	–
E	14.1	12.5	15.6	300	6.2	–	–
F	14.1	12.5	11.0	300	6.1	–	–
G	14.1	12.5	11.0	100	10	0.36	0.31
			15.6	300	5.2	0.14	0.13

Supplementary Table 2: Deconvolution and DNP enhancements (ε) of the ^{7}Li signals of microstructural lithium deposited from the LP30 and LP30+FEC electrolytes (samples D and E) at room temperature (Fig. 3a–d, main text), and for sample E at 105 K (Supplementary Fig. 14). See Supplementary Fig. 15 and Supplementary Fig. 16.

LP30 (300 K)				
Chemical shift /ppm	−0.8	−0.3	0.7	264
FWHM /ppm[a]	0.1	2.4	6.6	–
Integration[b]	11%	11%	78%	–
ε	0.95	4.8	8.1	13
**LP30 + FEC (300 K)**				
Chemical shift /ppm	−0.9	−0.8	0.1	264
FWHM /ppm[a]	0.3	1.5	6.4	–
Integration[b]	22%	13%	65%	–
ε	3.9	10.3	5.6	6.2
**LP30 + FEC (105 K)**				
Chemical shift /ppm	0.0	2.05	2.86	265
FWHM /ppm[a]	4.4	7.4	18	–
Integration[b]	15%	34%	51%	–
ε	0.95	5.8	4.8	4.6

[a]Full-width at half-maximum

[b]Relative integrated intensity of the diamagnetic signals in the microwave on spectra.

Supplementary Table 3: Deconvolution and DNP enhancements (ε) of the diamagnetic ^{7}Li signals (Supplementary Fig. 13b,d) of microstructural lithium deposited from the LP30 and LP30+FEC electrolytes (samples F and G). There is some additional error introduced to the fits due to field drift and an imperfect shim.

	LP30		LP30 + FEC		
Shift /ppm	−0.8	−0.2	−0.8	−0.6	−0.1
FWHM /ppm	0.18	4.5	0.35	2.3	8.5
Integration /%	45	55	14	17	69
ε	1.9	4.0	1.2	3.7	4.4

Supplementary Table 4: Deconvolution and DNP enhancements (ε) of the direct ^{1}H NMR spectra (Supplementary Fig. 13e,g) of microstructural lithium deposited using the LP30 and LP30+FEC electrolytes (samples F and G). The enhancements are calculated either from the ratio of the microwave off and on spectra, or from the intensity in the difference spectrum if the signal could not be easily distinguished in the microwave-off spectrum.

	LP30				LP30 + FEC			
Shift /ppm	1.32	3.8	4.6	4.73	1.33	4.48	4.65	7.23
FWHM /ppm	0.93	0.56	4.78	0.57	0.65	6.69	0.91	0.45
Integration /%	4.3	4.6	63.8	27.3	1.1	69.9	26.0	3.0
ε	2.0	∞[a]	1.0	1.7	~1.4 – 2.7	1.1	1.8	0.9

[a]This signal could not be seen in the microwave off spectrum, and the intensity in the difference spectrum is the same as in the microwave on spectrum.

Supplementary Table 5: Recycle delays, number of scans and experimental time for the experiments shown in Fig. 3 in the main text.

	Experiment	Recycle delay /s	Scans	Experimental time
a – d	Diamagnetic ^{7}Li	1	32	32 s
e	Direct ^{1}H, LP30	10	16	2 min 41 s
g	Direct ^{1}H, LP30+FEC	10	32	5 min 23 s
i	Direct ^{19}F, LP30	30	4	2 min
k	Direct ^{19}F, LP30+FEC	3	32	1 min 37 s
f, h, j, l	$^{7}Li \rightarrow ^{1}H$ and $^{7}Li \rightarrow ^{19}F$ CP	1	320	5 min 44 s

Supplementary Table 6: Deconvolution and DNP enhancements (ε) of the direct ^{1}H NMR spectra (main text, Fig. 3e,g, main text) of microstructural lithium deposited using the LP30 and LP30 + FEC electrolytes (samples D and E). The deconvoluted spectra are shown in Supplementary Fig. 17 and Supplementary Fig. 18. The enhancements are given by the ratio of the microwave off and on spectra; if the signal could not be easily distinguished in the microwave-off spectrum, the microwave-off intensity was calculated by subtracting the deconvoluted intensity in the difference spectrum from that of the microwave-on spectrum.

LP30								
Chemical shift /ppm	0.10	1.26	3.8	3.87	4.68	4.68	4.74	7.27
FWHM /ppm[a]	0.15	1.10	0.094	0.55	3.93	0.12	0.52	0.09
Integration /%[b]	2.50	3.84	0.29	2.26	68.66	6.55	13.82	2.07
ε	1.0	1.3	1.9	∞[b]	1.1	2.0	1.8	0.9
LP30 + FEC								
Chemical shift /ppm	0.17	1.00	1.39	1.70	2.14	4.50	4.71	7.22
FWHM /ppm[a]	0.5	0.28	0.22	0.2	0.32	7.58	1.82	0.37
Integration[b]	0.05	84.16	0.15	0.03	0.04	8.82	6.64	0.12
ε	1.1	1.2	1.5	1.5	1.4	1.0	1.3	0.7

[a]Full-width at half-maximum

[b]This signal could not be seen in the microwave-off spectrum, and the intensity in the difference spectrum is the same as in the microwave-on spectrum within error.

Supplementary Table 7: Summary of different microstructural lithium samples used in this work.

	Substr.	Electrolyte	Current density /mA cm^{-2}	Experiments
A	Li	LP30+FEC	1.25	^{7}Li metal spectra, RT and 100 K. RT field sweep.
B	Cu	LP30	0.033	100 K full field sweep.<br>Metal spectra as a function of microwave power.
C	Cu*	LP30	≤ 0.455	^{7}Li experiments at 9.4 T.
D	Li	LP30	1.25	Main text comparison of electrolytes.
E	Li	LP30+FEC	1.25	Main text comparison of electrolytes.<br>Enhancement as a function of T and power.
F	Li	LP30	0.5	SI comparison of electrolytes.
G	Li	LP30+FEC	0.5	SI comparison of electrolytes.<br>Field sweeps for different μw powers.

* NMC811 used as the Li source. All other samples used Li metal.

Supplementary Table 8: Skin depths for the radiofrequency and microwave penetration of Li metal.

Radiation	Frequency	Skin depth, 300 K	Skin depth, 100 K
Radiofrequency	233 MHz	8.6 μm	3.7 μm
Microwave	395 GHz	0.21 μm	0.09 μm

Supplementary Table 9: The increase in sample temperature ΔT on microwave irradiation at room temperature as measured by the change in the chemical shift at the peak intensity and of the centre of mass of the (C.O.M) of the KBr ^{79}Br resonance, as well as the full-width at half-maximum (FWHM), for microstructural lithium mixed with KBr and for pure KBr, at 12.5 kHz MAS.

		Peak intensity /ppm	ΔT /K	C.O.M /ppm	ΔT /K	FWHM /ppm
Li/KBr	μw on	0.66	31	0.50	34	1.67
	μw off	1.44		1.35		1.34
KBr	μw on	0.65	36	0.65	35	1.22
	μw off	1.55		1.53		1.10

Supplementary Note 1: Skin Depth Calculation

The skin depth for penetration of electromagnetic radiation into a metal is given by:[6]

$$d = \sqrt{\frac{\rho}{\pi \mu_0 \mu_r \nu}} \tag{1}$$

where ρ the resistivity of the metal (92.8 nΩ m for Li metal at 293 K[7]), μ_0 is the vacuum permeability, μ_r is the relative permeability (1.4 for Li metal[7]) and ν is the frequency of the radiation.

For the radiofrequency and microwave radiation used at 14.1 T in this study, this yields the skin depths shown in Supplementary Table 8. At lower temperatures the resistivity decreases, resulting in a shallower skin depth.

Supplementary Note 2: Microwave-induced Sample Heating

In order to investigate the degree of sample heating induced by microwave irradiation at room temperature, the ^{79}Br spectra were recorded for a microstructural lithium sample mixed with KBr and for pure KBr, both with and without microwave irradiation (Supplementary Fig. 33). The ^{79}Br resonance in KBr has a temperature dependence of −0.025 ppm/K.[8] Firstly, under MAS the microstructural lithium sample has a higher sample temperature than pure KBr by ~4 K, which could be due to eddy currents in the metal. Under microwave irradiation the heating for each sample is similar with the temperature increasing by ~35 K (Supplementary Table 9), although the nominal temperature increase as measured by the sample thermocouple was only 8 K. The linewidth is greater for the microstructural lithium sample, however, suggesting a broader distribution of sample temperatures; in particular, on microwave irradiation the resonance spreads to even lower frequencies, suggesting there could be hot spots in the sample which experience greater heating. It is challenging to determine the extent of the temperature variations within the sample since there is also an inherent contribution to the linewidth of the ^{79}Br signal, particularly for the heterogeneous lithium sample, but by comparing the full-width at half-maximum of the resonances, the microstructural lithium sample may experience a temperature range of ~20 K. The sample heating appears to be more severe at room temperature than at 100 K, where sample heating was approximately 5 K for a pure KBr sample.

Supplementary Methods

Electrochemistry

Copper metal disks (MTI) were soaked in concentrated acetic acid for 10 minutes for oxide removal. The acetic acid residue was removed with dry nitrogen flow and the disks were dried at 100 °C under vacuum overnight. The lithium metal disks (LTS research, 99.95%) were used without any pretreatment. Printed $LiNi_{0.8}Mn_{0.1}Co_{0.1}O_2$ (NMC811) electrodes were fabricated in Argonne National Laboratory (A-C020, made by CAMP facility) using the following materials: 90 wt % NMC811 (Targray), 5 wt % conductive carbon (Timcal C45), and 5 wt % PVDF binder (Solvay 5130). They were dried overnight at 120 °C under dynamic vacuum.

Li–Li, Li–Cu and NMC811–Cu coin cells were assembled in an Ar atmosphere glovebox (O_2, H_2O < 1 ppm, MBraun) in stainless-steel 2032 coin cells (Cambridge Energy Solutions) with a stainless-steel conical spring, two 0.5 mm thick stainless-steel spacer disks and polypropylene-polyethylene separator (Celgard 3501, dried under vacuum at 40°C). For NMC811–Cu cells, an additional glass fibre separator (GF/B, Whatman) was used on the NMC811 side. The coin cell components were dried for at least a week at 60 °C. The electrolyte consisted of 75 μL of 1 M $LiPF_6$ in 1:1 v/v ethylene carbonate/dimethyl carbonate (EC:DMC; Sigma Aldrich, battery grade), referred to as LP30, and the same electrolyte with 1:10 volume ratio of the additive fluoroethylene carbonate (FEC; Sigma Aldrich, anhydrous), referred to as LP30+FEC.

Galvanostatic electrodeposition was performed using a Biologic MPG2 battery cycler with EC-Lab software. For all the samples except C, the constant currents shown in Supplementary Table 7 were applied in a single direction for a capacity of 4 mAh cm^{-2} (e.g. Supplementary Fig. 30). For sample B, constant current was applied at 0.033 mA cm^{-2} for 60 hours (2 mAh cm^{-2}) followed by 12 cycles of stripping for 1 hour and plating for 5 hours (Supplementary Fig. 31), to increase the amount of the SEI formed. For sample C, galvanostatic charging was performed at 0.455 mA cm^{-2} until the voltage reached 4.5 V, followed by holding at 4.5 V for 8 hours; over both steps, a total charge of 1.76 mAh cm^{-2} was transferred (Supplementary Fig. 32). The coin cells were disassembled in an Ar atmosphere glovebox and the microstructures scraped off gently with a razorblade without rinsing. The sample was then diluted by ~5× by mass with dry KBr by lightly hand-grinding with an agate mortar and pestle to improve microwave penetration and allow the metallic samples to be more easily spun; this does not have a significant effect on the microstructural morphology (see SEM images, Supplementary Fig. 28). The sample was finally packed into a 3.2 mm outer diameter (2.2 mm inner diameter, 28 μl volume) sapphire rotor.

NMR Spectroscopy

For the NMR experiments performed at 14.1 T, radiofrequency (rf) powers were used of 45 kHz for ^{7}Li, 110 kHz for ^{1}H and 85 kHz for ^{19}F. For the CP experiments, rf powers during contact were used of ~45 kHz for ^{7}Li , ~85 kHz for ^{1}H and ~70 kHz for ^{19}F. The experiments at 9.4 T used a ^{7}Li rf power of 100 kHz. ^{1}H NMR spectra were referenced to adamantane at 1.81 ppm, ^{7}Li spectra to LiF at −1 ppm and ^{19}F spectra to LiF at −203 ppm, all at room temperature. The gyrotron microwave powers were measured using a calorimeter half-way along the waveguide, while the klystron power was measured at the output using a directional coupler then scaled to account for microwave reflection. Spectra were deconvoluted using dmfit software.[9]

ESR Spectroscopy

Continuous-wave X-band ESR measurements were performed on pieces of lithium metal on a Bruker E500 X-band spectrometer with an ER 4122SHQE cavity, tuned to 9.373 GHz. The external magnetic field was modulated at 100 kHz with a modulation amplitude of 1 G. The power saturation experiment was performed on a Bruker EMX Plus X-band ESR spectrometer.

SEM

SEM images were taken with a Tescan MIRA3 FEG-SEM instrument at an acceleration voltage of 5.0 kV. The coin cells were disassembled in an Ar glovebox and the unrinsed electrodes were mounted onto the SEM stage of an air-sensitive transfer module (Kammrath & Weiss, type CT0) and dried under vacuum. The samples were then transferred into the SEM chamber using the air-sensitive transfer module under an inert Ar atmosphere. No post-processing was applied to the images.

Supplementary References

1. Overhauser, A. W. Polarization of Nuclei in Metals. *Phys. Rev.* **92**, 411–415 (1953).
2. Overhauser, A. W. Paramagnetic relaxation in metals. *Phys. Rev.* **89**, 689–700 (1953).
3. Korringa, J. Nuclear magnetic relaxation and resonnance line shift in metals. *Physica* **16**, 601–610 (1950).
4. Altenbach, C., Froncisz, W., Hemker, R., Mchaourab, H. & Hubbell, W. L. Accessibility of Nitroxide Side Chains: Absolute Heisenberg Exchange Rates from Power Saturation EPR. *Biophys. J.* **89**, 2103–2112 (2005).
5. Ilott, A. J. & Jerschow, A. Probing Solid-Electrolyte Interphase (SEI) Growth and Ion Permeability at Undriven Electrolyte–Metal Interfaces Using ^{7}Li NMR. *J. Phys. Chem. C* **122**, 12598–12604 (2018).
6. Kittel, C. *Introduction to Solid State Physics*. (John Wiley & Sons, 2004).
7. Lide, D. R. *CRC Handbook of Chemistry and Physics*. (CRC Press, 2003).
8. Thurber, K. R. & Tycko, R. Measurement of sample temperatures under magic-angle spinning from the chemical shift and spin-lattice relaxation rate of ^{79}Br in KBr powder. *J. Magn. Reson.* **196**, 84–87 (2009).
9. Massiot, D. *et al.* Modelling one- and two-dimensional solid-state NMR spectra. *Magn. Reson. Chem.* **40**, 70–76 (2002).
